# Nascent RNA signaling to yeast RNA Pol II during transcription elongation

**DOI:** 10.1371/journal.pone.0194438

**Published:** 2018-03-23

**Authors:** Eva Klopf, Murielle Moes, Fabian Amman, Bob Zimmermann, Frederike von Pelchrzim, Christina Wagner, Renée Schroeder

**Affiliations:** 1 Max F. Perutz Laboratories (MFPL); University of Vienna; Vienna, Austria; 2 Institute for Theoretical Chemistry; University of Vienna; Vienna, Austria; 3 Department of Molecular Evolution and Development; University of Vienna; Vienna, Austria; Texas A&M University, UNITED STATES

## Abstract

Transcription as the key step in gene expression is a highly regulated process. The speed of transcription elongation depends on the underlying gene sequence and varies on a gene by gene basis. The reason for this sequence dependence is not known in detail. Recently, our group studied the cross talk between the nascent RNA and the transcribing RNA polymerase by screening the *Escherichia coli* genome for RNA sequences with high affinity to RNA Pol by performing genomic SELEX. This approach led to the identification of RNA polymerase-binding APtamers termed “RAPs”. RAPs can have positive and negative effects on gene expression. A subgroup is able to downregulate transcription via the activity of the termination factor Rho. In this study, we used a similar SELEX setup using yeast genomic DNA as source of RNA sequences and highly purified yeast RNA Pol II as bait and obtained almost 1300 yeast-derived RAPs. Yeast RAPs are found throughout the genome within genes and antisense to genes, they are overrepresented in the non-transcribed strand of yeast telomeres and underrepresented in intergenic regions. Genes harbouring a RAP are more likely to show lower mRNA levels. By determining the endogenous expression levels as well as using a reporter system, we show that RAPs located within coding regions can reduce the transcript level downstream of the RAP. Here we demonstrate that RAPs represent a novel type of regulatory RNA signal in *Saccharomyces cerevisiae* that act in *cis* and interfere with the elongating transcription machinery to reduce the transcriptional output.

## Introduction

Transcription of mRNA is a highly regulated process influenced by numerous protein factors at each stage of the transcription cycle [1–7]. RNAs have also been shown to affect transcription in various ways. Non-coding RNAs (ncRNAs) in yeast and mammals have been shown to regulate chromatin features, promoter proximal pausing of RNA Pol II or to facilitate DNA looping [8,9]. In addition, ncRNAs can build a platform for protein factor association [10] to RNA Pol II or occlude transcription factor binding [11]. Recently, also nascent RNA itself has been proven to be directly involved in regulation of transcription by trapping transcription factors at the promoter thereby producing a positive feedback loop [12]. Moreover, nascent RNA contains regulatory elements that cross-talk with RNA Pol II via RNA-binding factors such as Nrd1 and Nab3 in yeast [13]. Thus, the concept that the nascent RNA influences its own expression via the interaction with protein factors is well documented.

Only a few *trans*-acting RNAs have been reported to directly bind to RNA polymerases and modulate its activity: 6S RNA (*E*. *coli*), B2 RNA (*M*. *musculus*), and Alu RNAs (*H*. *sapiens*) all inhibit transcription at the stage of initiation [14–18]. Moreover, an *in vitro* selected artificial RNA, the FC aptamer, is able to exhibit inhibition of transcription of yeast RNA Pol II by binding to the active center cleft [19,20]. In prokaryotic systems, the highly-structured *putL* and *putR* RNA elements interact with the beta-prime subunit of RNA polymerase thereby inducing transcription antitermination [21,22].

In a recent publication, our group showed for the first time that nascent RNA is able to directly regulate RNA polymerase activity in *E*. *coli*. A combination of SELEX and Next Generation Sequencing led to the identification of sequences within the nascent transcripts, termed RAPs (RNA polymerase binding APtamers), that show a very high affinity to RNA polymerase. A subgroup of RAPs was identified to exert a negative effect on transcription. These iRAPs (inhibitory RAPs) can downregulate elongation in *cis* via combined mechanisms mediated by the bacterial termination factor Rho [23].

Here, we addressed the question whether specific sequences in the nascent RNA can interact with RNA polymerase II and modulate transcription in *cis* in the budding yeast *S*. *cerevisiae*. We chose a similar strategy as used for *E*. *coli* aiming to identify RNA elements that have a high affinity to RNA Polymerase II (RNA Pol II) using a genomic SELEX screen. This approach allowed us to identify almost 1300 yeast RAPs. We show that RAP activity is not only restricted to prokaryotes but can also affect eukaryotic RNA polymerase II. RAPs in *S*. *cerevisiae* represent *cis*-acting regulatory RNA signals that can interfere with the elongation machinery possibly causing premature intragenic termination of transcription.

## Results

### Genomic SELEX identifies RAPs encoded in the yeast genome

**Fig 1:**
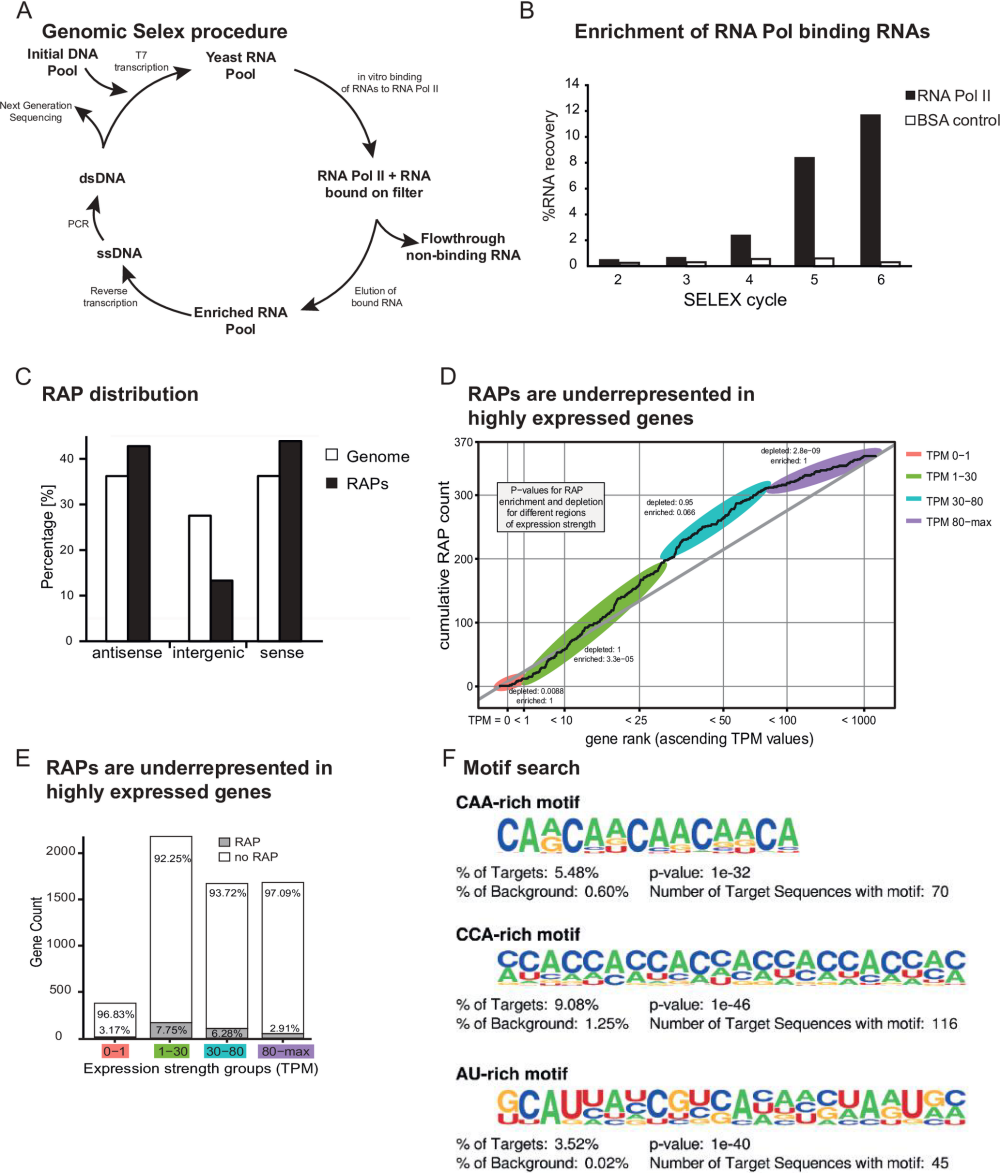
Genomic SELEX RNA Pol II binding RNAs. (A) Schematic outline for our approach to isolate novel RNA Pol II-binding RNAs. After construction of a genomic library via random priming, DNA fragments are *in vitro* transcribed and subjected to multiple rounds of SELEX to isolate RNA Pol II-binding RNAs. (B) Enrichment profile for the selection of RNA Pol II-binding RNAs, showing the percentage of recovered RNA per cycle. RNAs were selected via filter binding. Low stringency conditions (10μM RNA and 1μM protein) were applied for the six consecutive cycles to firmly establish a population of binding species. Since a 10-fold molar excess of RNA over protein was used to support competition, these values correspond to 100% of the total amount of RNA able to bind RNA Pol II, assuming a single binding site on the protein. (C) After deep sequencing, the enriched RNA domains (~1300), termed RAPs (RNA polymerase-binding aptamers) were mapped to the yeast genome and classified according to their genomic location. Percentage of RAPs classified as being intergenic, sense or anti-sense to annotated genes, compared to the relative occurrence of these classes in the yeast genome. (D) Analysis of expression levels of RAP-containing genes. Only RAPs oriented in sense to open reading frames were analyzed (total 370). On the x-axis the genes are ordered according to their transcript abundance (TPM = transcripts per million) [62]. On the y-axis the cumulative count of genes including a RAP up to the corresponding TPM is plotted. If RAPs were uniformly distributed over all genes irrespective of their transcript abundance the curve should follow the straight diagonal. Genes in segments with a reduced slope are depleted of RAPs, genes in segments with a larger slope than the diagonal are enriched in RAPs. Different segments were tested for a significant difference in RAP density to the expected uniform distribution using a Poisson test. (E) Bar graph showing the number of genes in each category and the percentage of RAPs in each category. (F) Analysis for overrepresented sequence motifs in RAPs compared to the genomic background. The three best ranked motifs according to their p-values are shown.

In order to discover genomic encoded RNAs which might be involved in transcription regulation in *S*. *cerevisiae*, we chose the unbiased approach of Genomic SELEX (Systematic evolution of ligands by exponential enrichment). We constructed an initial library starting from short (30-400nt) fragments of the yeast genome to screen for RNAs binding with high-affinity to a complete purified yeast RNA Pol II 12 subunit complex. In course of the abovementioned SELEX procedure [24,25] (Fig 1A) RNAs started to enrich during the 4^th^ cycle (Fig 1B) showing that the vast majority of the RNA starting pool does not bind to RNA Pol II. After six rounds of SELEX the majority of enriched RNAs are bound to RNA Pol II (ratio RNA:protein 10:1 to increase selectivity). These enriched sequences from round 6 were subjected to Illumina sequencing for further characterization, resulting in 2,760,203 sequenced reads mapping to the yeast genome. The enriched sequences were defined as RAPs. The complete list of the 1300 identified yeast RAPs can be found in S1 Table.

RAPs were characterized according to various attributes: genomic location, expression levels of the host gene, functional gene group and potential common sequence motifs. RAPs were found to be underrepresented in intergenic regions relative to annotated genes and similarly distributed among the sense and antisense strand (Fig 1C). This finding is in contrast to *E*. *coli* RAPs, which are preferentially oriented in antisense [23]. Interestingly, a more detailed analysis for localization in different genetic elements revealed that RAPs are significantly enriched in rDNA, telomeric and subtelomeric regions (S1A Fig). Next, we asked for the transcript abundance levels of RAP-containing genes by exploring a published RNA-seq data set [26]. We focused on genes with RAPs located sense to open reading frames and sorted them according to their expression strength (TPM, transcripts per million). Categorization in four manually defined segments (TPM<1; 1<TPM<30; 30<TPM<80; 80<TPM<max) revealed that sense RAPs are underrepresented in genes with very lowly (TPM<1; p-value 8.8*10^−3^) and very highly abundant transcripts (TPM>80; p-value 2.9*10^−9^). Importantly, sense RAPs were found to be enriched in genes with low transcript levels (1<TPM<30; p-value 3.3*10^−5^) (Fig 1D and 1E). We further performed a gene ontology term enrichment analysis and found that RAP-containing genes are enriched in the group of genes that are involved in regulation of transcription (S1B and S1C Fig).

Next, we examined if yeast RAPs share a common sequence motif that might be responsible for RNA Pol II binding activity. The length of the enriched sequences ranges from 40 to 400 bases and they do not display any significant differences in nucleotide content. A detailed search among RAPs using the Homer software [27] showed that they do not share a single common sequence motif. Most of the identified enriched motifs are characteristic for a small subset of RAP sequences and display a rather low specificity. However, we found a subset of RAPs containing low complexity sequence repeats which are either CAA-, CCA-, or AU-rich and vary between 14 (CAA-rich) to 22 nucleotides (CCA and AU-rich) in length (Fig 1F). These findings suggest that RAPs are highly diverse in their sequences which mediate the interaction with RNA Pol II.

### RAPs can down-regulate elongation

To directly test whether yeast RAPs can have an impact on transcription we developed a reporter system that enabled comparative analysis of any representative RAP sequence or sequence variant in a context-independent manner. In short, a reporter construct consisting of a *GFPURA3STOPHIS3* fusion under the control of an inducible *GAL1-10* promoter was created. This fusion construct enabled recombination of the sequence of interest between *GFP* and *HIS3* (for details see S2A Fig and Materials and Methods). To ensure that the *GFPHIS3* fusion transcript displays equally distributed signal intensities we analyzed expression levels using qRT-PCR. Strain EKYGH carrying the *GAL1-10prGFPHIS3* construct was grown on medium containing 2% galactose to early logarithmic phase and RNA was isolated, DNAse I digested, and transcript levels were analyzed with amplicons indicated in S2B Fig. We detected similar expression levels all over the fusion transcript (S2C Fig). Next, we checked for full activation of the *GAL1-10* promoter in this genomic context and performed a time-course experiment. Transcript signals were clearly detectable 10 minutes after addition of 2% galactose to cells grown on 2% raffinose and reached maximum levels after 60 minutes similar to endogenous *GAL1-10* transcripts [28–30] (S5B Fig).

**Fig 2:**
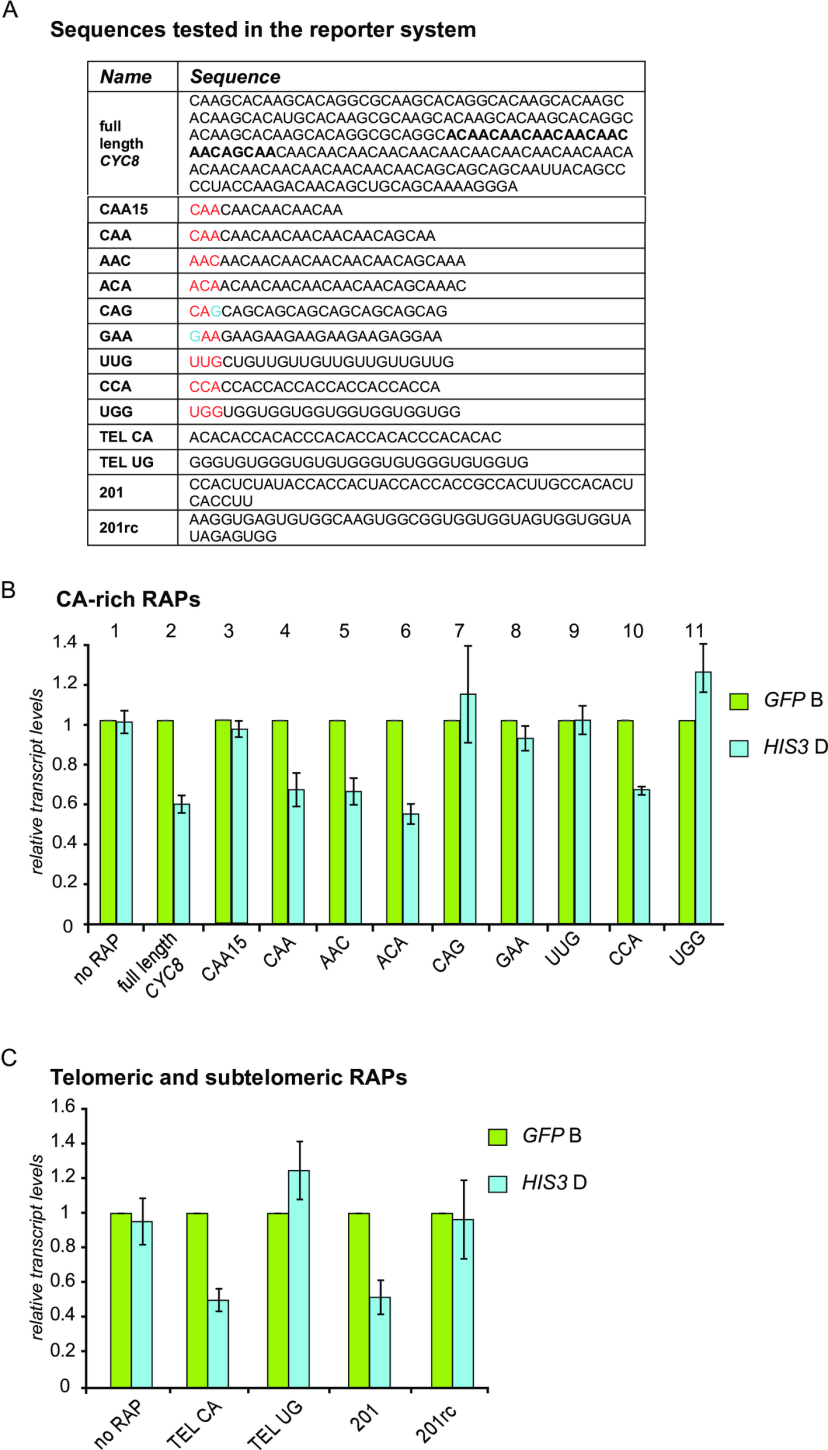
Sequence requirements for RAP activity. (A) Sequences tested in the reporter system (For details see S5 Table). (B) Cells containing reporter constructs (listed in 2A) were grown in medium with 2% galactose and transcript levels were determined as described in Materials and Methods using primer pairs shown in S2B Fig. The minimal CAA-rich consensus motif of 15bp (lane 3) does not show any effect on expression levels. The CAA sequence of 24bp (lane 4) and the variants AAC (lane5) and ACA (lane6) found in *CYC8* and *CBK1* show a 40% decrease of *HIS3* transcript levels. Mutation of CAA to CAG or GAA alleviates the decrease (lanes 7 and 8). The CCA repetitive motif also has a negative effect on elongation (lane 10). The reverse complement control sequences UUG (lane 9) and UGG (lane 11) do not show any changes in *HIS3* expression levels. (C) Telomeric and subtelomeric RAPs also have a negative effect on elongation. Cells carrying telomeric and subtelomeric RAP sequences (as listed in A) were treated similarly to (B) The reverse complement control sequences do not show any effects.

After verification of the full functionality of the reporter construct we tested the effect of a RAP sequence on the expression levels of the reporter system. We chose the full length RAP133 (240bp) of the *CYC8* locus as an initial candidate (Fig 2A) since it has a moderate read count in the SELEX approach and contains multiple repeats of the CAA-rich consensus motif identified in our Homer search (Fig 1F). To investigate whether the promoter activity has any impact on the potential RAP activity we performed a dynamic approach and analyzed expression levels of cells grown in raffinose and shifted to galactose similar as in S2D Fig. We detected a 40% drop in *HIS3* transcript levels compared to GFP levels (S2E Fig) at all analyzed time points (Raff, 10, 30, and 60min) in cells with the RAP-containing constructs. Therefore, the full length RAP133 of *CYC8* has a negative impact on elongation in the reporter construct independent of promoter activity. These results suggest that RAPs in *S*. *cerevisiae* are RNA sequences that can interfere with transcription elongation similar to iRAPs identified in *E*. *coli*. In the following we will refer to this observed reduction of transcript levels as “RAP effect”.

We used the *Gal1-10prGFPHIS3* reporter construct to determine the minimal RAP size necessary to down-regulate elongation and to specify the sequence requirements by performing mutational analyzes. First, we introduced the identified minimal consensus motif obtained from our search with Homer software (CAA-rich, 15bp) (Fig 2A). This minimal sequence did not show an effect on *HIS3* expression levels (Fig 2B, compare lane 1 and 3) and thus, might be simply too short for interfering with elongation. Therefore, we introduced the highly-enriched region of the *CYC8* RAP133 into the reporter. This sequence of 24bp represents that particular region of the identified RAP that shows the highest read count derived from the genomic SELEX sequencing. This sequence had a similar repressive effect (~40% drop) on *HIS3* expression as the full length RAP133 of *CYC8* (Fig 2B, compare lanes 1, 2 and 4). Importantly, the reverse complement control sequence did not cause any decrease in transcript levels of *HIS3* (Fig 2A and 2B, compare lanes 1, 4 and 9).

### RAPs act at the RNA level and are independent of the encoded protein and double stranded DNA sequence

From the previously obtained data we could not exclude the possibility that the RAP effect was caused by the underlying double stranded DNA sequence of the host gene. Therefore, we tested whether the reading frame, and thus the protein sequence of the host gene, might play a role. Changing the sequence from CAA to AAC or ACA repeats completely altered the amino acid composition of the encoded proteins but did not alleviate the repressive RAP effect (Fig 2B, compare lanes 1, 4, 5 and 6). In contrast, mutation of the wobble base from A to G completely restored *HIS3* expression levels without changing the amino acid composition (Fig 2B, lane 7). Similarly, changing the repetitive sequence from CAA to GAA resulted in equal *GFP* and *HIS3* expression levels (Fig 2B compare lane 1, 4 and 8).

The second motif identified by Homer displayed a CCA-rich sequence and therefore was also tested in the reporter together with its reverse complement control (Fig 2A). This motif variant also caused a ~40% reduction of *HIS3* expression, and importantly, the reverse complement control did not (Fig 2B, lanes 1, 10 and 11). These results show that a RAP sequence as short as 24nts is sufficient to reduce transcript levels downstream of the RAP. Furthermore, the protein sequence encoded by the RAP has no impact on transcription elongation. Most importantly, the reverse complementary control sequences showed similar *GFP* and *HIS3* transcript levels and therefore it is very unlikely that the underlying RAP encoding double stranded DNA sequence is a simple obstacle for the elongation complex.

### Telomeric and subtelomeric RAPs cause reduction of transcriptional elongation

We found a significant enrichment of RAPs on the CA-rich strand of yeast telomeres with a motif consensus 5’-(C_1-3_A)n-3’ (enrichment 136.1; p-value<10^−5^). This enrichment was almost two times higher than for the GU-rich strand (enrichment 76.977; p-value<10^−5^), known to be transcribed in yeast forming the long non-coding RNA termed TERRA ([31] S1A Fig). Yeast telomeres are transcribed in a strand-specific manner starting at the subtelomeric region of the chromosome and terminating within the telomeric repeat tract (C1-3A/TG1-3) [32–34]. The transcriptional regulation of yeast telomeres has been studied in detail but it has neither been determined what leads to the low expression levels of the TERRA strand nor to the complete silencing of its reverse complement ARIA strand [32–35]. To test whether these sequences influence elongation we recombined 30bp of the C-rich and G-rich telomere ends into the *GFPHIS3* reporter system (Fig 2A). As expected, the RAP derived from the telomeric C-rich strand caused a repressive effect on *HIS3* expression (50%) whereas the G-rich sequence did not (Fig 2C). The subtelomeric CCA-rich RAP201 was found in several regions in the genome (Chr XIII: 5895–5970, Chr IV: 459–636, Chr XVI: 942565–942832, Chr II: 812669–812832). Therefore, this subtelomeric RAP was an additional interesting candidate to test in our reporter system (Fig 2A). RAP201 reduced *HIS3* expression by ~40% (Fig 2C). These results indicate that RAPs derived from telomeric and subtelomeric regions can also cause a reduction in transcriptional elongation.

### RAPs can down-regulate transcription elongation of their host mRNAs

**Fig 3:**
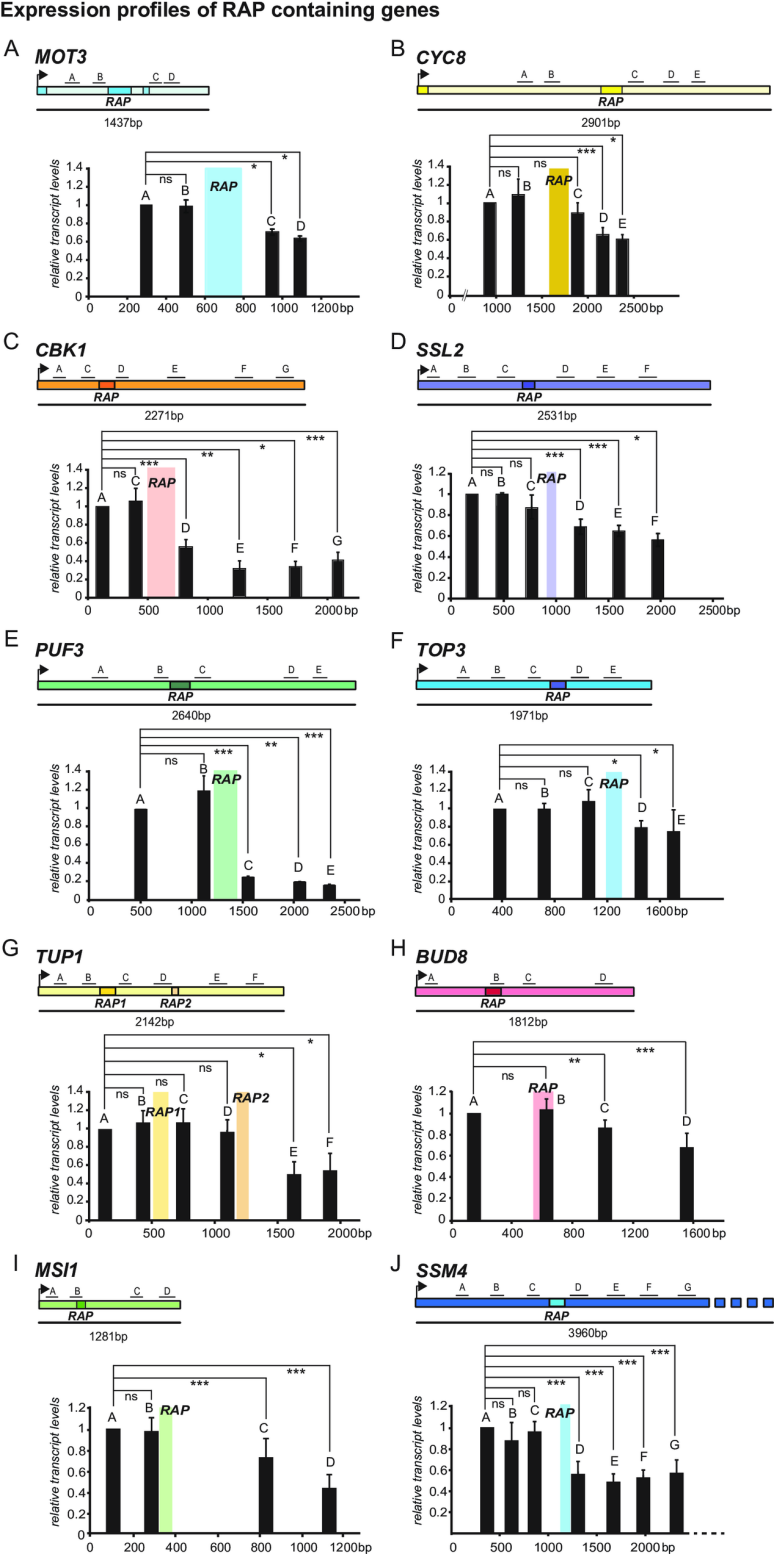
Expression profiles of RAP-containing genes. (A) to (J) Transcript levels of 10 selected target genes (*MOT3*, *CYC8*, *CBK1*, *SSL2*, *PUF3*, *TOP3*, *TUP1*, *BUD8*, *MSI1* and *SSM4* respectively). Yeast cells were grown to exponential phase, total RNA was extracted and DNAseI digested. Reverse transcription was performed, and expression levels were quantified by qRT-PCR using amplicons upstream and downstream of RAPs as indicated in the respective figures. Values were normalized to the first 5’ amplicon and represent means ° SD n ≤ 3; ***p<0.001; **p<0.01; *p≤0.05; ns, not significant (tested by t-test).

The results obtained from our reporter construct tempted us to ask whether RAPs can have a direct impact on the transcription of their host mRNAs and therefore we used a similar approach to quantify mRNA levels of endogenously expressed genes along their sequence. We performed qRT-PCRs using multiple amplicons located upstream and downstream of the endogenous RAPs. The candidate genes were chosen according to three basic criteria: orientation of the RAP in sense, location of the RAP in the middle of the host mRNA, avoiding the very 5’ and 3’ ends, and relatively robust expression levels to facilitate detection. Specifically, the analyzed candidate genes were *CYC8*, already analyzed in the reporter, *MOT3* due to the extremely high read count of RAP1027, *CBK1*, *PUF3* and *TOP3* containing the CAA-rich sequence identified by motif search and tested in the reporter, *TUP1*, the protein interaction partner of *CYC8*, and seven randomly chosen genes (*SSL2*, *MSI1*, *BUD8*, *SSM4*, *TUB4*, *PRY3* and *SRV2*) (S2 Table). Exponentially growing BY wild type cells (S3 Table) were harvested, total RNA was isolated, and DNA was digested. cDNA was primed using random nonamers and qRT-PCR was performed. Of the 14 tested candidate RAPs (*TUP1* contains 2 sense and 1 overlapping antisense) 10 show a significant drop in transcript levels downstream of the RAP. The reduction ranged from ~20% (*TOP3*, *BUD8*, Fig 3F and 3H) to 80% (*PUF3*, Fig 3E). Interestingly, of the two *TUP1* RAPs only the region containing an overlapping sense and antisense RAP (sense RAP180 and antisense RAP194) shows an effect on expression levels (Fig 3G). RAPs in the *TUB4*, *PRY3* and *SRV2* genes did not cause any reduction in transcript levels (S4G, S4H and S4I Fig). However, we cannot exclude that these RAPs have other effects on transcription not detectable in our experimental setup.

We analyzed the transcriptional landscape of the 10 candidates that showed an effect in our qRT-PCR analysis to ensure that neither antisense transcription, nor products of cryptic and pervasive transcription (such as Cryptic Unstable Transcripts-CUTs and stable unannotated transcripts-SUTs) could interfere with our approach. We used the available data set derived from high resolution tiling microarrays by Xu et al. [36] including genome wide analysis of the transcriptome in BY wt and the exosome mutant *rrp6*Δ. This data set enables the detection of CUTs which were initially observed in this mutant background. S3 Fig shows browser screenshots of the chosen RAP-containing targets. We could not observe any transcript species that could interfere with our qRT-PCR approach. Therefore, the observed expression profiles are not simply a result of antisense or CUT/SUT transcription.

To confirm that the reduction in transcript levels is specific for RAP-containing genes we analyzed six control genes of varying expression levels for which no RAPs had been detected and did not observe any reduction in transcript levels towards the 3’ end of the genes (S4A–S4F Fig). These results indicate that RAPs can also affect transcription elongation in their endogenous context and that RAPs identified in *S*. *cerevisiae* can be responsible for *cis*-active inhibition of transcription.

**Fig 4:**
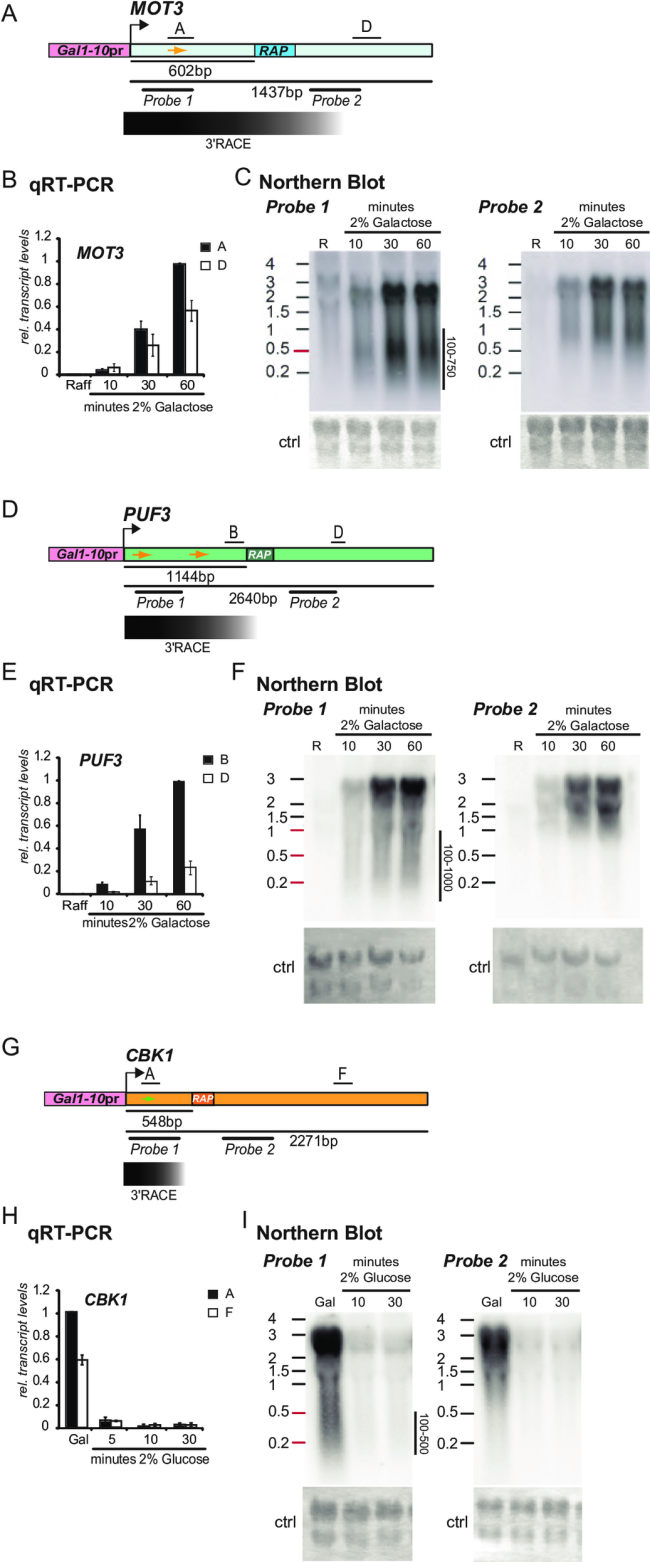
RAP-containing genes display multiple transcript species. (A) Schematic of position of amplicons for qRT-PCR and Northern Blot probes at the *MOT3* locus under the control of the *GAL1-10* promoter. The position of the specific forward primer used for 3’RACE is indicated as orange arrow. Distribution of transcript lengths determined by 3’ RACE is shown below. (B) qRT-PCR analysis of the *MOT3* transcript induced via the *GAL1-10* promoter. Cells grown on raffinose were treated with 2% galactose and aliquots were taken after 10, 30 and 60 minutes. A decrease of transcript levels downstream of the RAP can be detected at the 30 and 60 min. time points. (C) Northern Blot of the same samples as analyzed by qRT-PCR. The position of the probes is indicated in (A). *Probe1* (upstream of RAP) allows detection of short transcripts with a size between 100 and 750bp, the majority having a size of 500bp. With *probe2* these short transcripts are not detectable. (D) to (F) Similar to (A) (B) (C) but for the *PUF3* locus. (G) Position of amplicons for qRT-PCR and Northern Blot probes of the *CBK1* locus under the control of the *GAL1-10* promoter. The position of the specific forward primer used for 3’RACE is indicated as green arrow. Transcript ends determined by 3’ RACE are shown below. (H) Quantification by qRT-PCR shows a decrease of transcript levels downstream of the RAP. Cells were grown in medium containing 2% galactose. During exponential growth phase glucose was added to a final concentration of 2% and aliquots were taken after 5, 10 and 30 minutes to follow down-regulation of the promoter. (I) Northern Blot of the same samples (except the 5min. time point) analyzed with qRT-PCR. Short transcripts are detectable when *probe1* but not *probe2* is used.

The results obtained from our qRT-PCR analysis implies that RAP-containing genes might display at least two types of transcripts: A full length mRNA and a shorter species possibly ending in close proximity to the RAP. To test this assumption and to confirm the results of our qRT-PCR analysis, we performed Northern Blots of our candidate genes. Unfortunately, the obtained signals were too weak for a clear interpretation, possibly due to low expression levels and/or RNA instability. Therefore, we created strains carrying alleles of *MOT3*, *CBK1* and *PUF3* under the control of the inducible, highly active *GAL1-10* promoter (Fig 4A, 4D and 4G, S3 Table). We took advantage of the potential dynamic activation of this promoter and performed time course experiments to ensure specificity of the obtained signals. In addition, this approach enabled analysis of the endogenous RAP effect in a different promoter context. The strains Gal1prMOT3 and Gal1prPUF3 were grown on medium containing raffinose as the sole carbon source to early exponential phase and 2% galactose was added to induce the *GAL1-10* promoter. Aliquots were taken at the indicated time points. First, we analyzed expression levels by qRT-PCR and found a drop of transcript levels downstream of the RAPs recapitulating the results shown above and confirming that endogenous RAPs are also active under the inducible *GAL1-10* promoter (Fig 4B and 4E, compare with Fig 3A and 3E). Next, we performed Northern Blots with the same samples analyzed by qRT-PCR. We used two different probes for transcript detection, one located upstream (*probe1*) and one located downstream of the RAP (*probe2*) (Fig 4A and 4D). Both probes allowed for detection of specific full-length transcripts visible 10 minutes after addition of galactose and a maximum signal after 60 minutes. By using *probe1* we could clearly detect shorter transcripts for both genes, *MOT3* (~100-750bp) and *PUF3* (~100-1000bp). These signals were not detectable when *probe2*, located downstream of the RAPs, was used (Fig 4C and 4F). We performed a slightly different setup for the essential *CBK1* gene. Here we grew strain Gal1prCBK1 in medium containing galactose and added 2% glucose to repress the *GAL1-10* promoter. qRT-PCR also shows a drop of expression levels downstream of the RAP at all detectable time points (Fig 4H). The *CBK1* locus also displayed shorter transcripts hybridizing to *probe1* located upstream of the RAP in the Northern Blot (Fig 4I). These results show that RAP-containing genes display at least two different transcript species. To confirm that the observed short transcript species are specific for RAP-containing genes we analyzed the profile of the *GAL1* transcript under the control of its endogenous promoter by Northern Blotting. We performed a similar approach as for *MOT3* and *PUF3*. The probes were located at the 5’ end (*probe1*) and in the middle of the locus (*probe2*) (S5A Fig). Both probes allowed for detection of a single transcript of ~1500bp in size. This result indicates that multiple transcript species are a specific feature of the analyzed RAP-containing genes.

The size of the RNAs detected by Northern Blotting indicated that the majority of the short transcripts ended upstream of the RAP sequence. To map their 3’ ends we performed 3’RACE using adapter-labeled RNA (see materials and methods). The PCR products derived from gene-specific forward and adapter-specific reverse primers did not appear as a sharp, single band but as a smear reflecting multiple, short transcript species. The length distribution of these PCR products corresponded to the lengths of the short transcripts observed in the Northern Blots (Figs 4A, 4D, 4G and S6A–S6C). Thus, the majority of the short transcripts end upstream of the RAPs, however some species end in close proximity or even downstream of the RAP. Therefore, RAPs lead to disruption of transcription leading to short transcripts displaying undefined 3’ends.

### The nuclear exosome (RRP6), components of termination pathways (Nrd1 and Rat1) and the elongation factor (TFIIS) do not contribute to the RAP effect

The fact that the identified short transcripts end upstream of the RAPs and do not appear as a clear, single band but rather as a smear can have many reasons. It could reflect an RNA Pol II “traffic jam” preceding the RAP, but also an inherent instability of the transcript at the 3’ end similar as observed for other transcript species such as CUTs. The nuclear exosome has been shown to be involved in the degradation of aberrant mRNAs and products of pervasive transcription [36–41]. To test whether the identified short transcripts derived from RAP-containing genes are substrates of the nuclear exosome and thus, display characteristics of CUTs, we used a mutant strain lacking the intranuclear exosome subunit RRP6 in the Gal1prMOT3 strain background and performed Northern Blotting and qRT-PCR. We undertook a similar experimental setup as in Fig 4A, 4B and 4C. Unexpectedly, we could not detect any significant difference in the *MOT3* expression pattern between wild type and *rrp6*Δmutant. The short transcripts detected by *probe1* displayed a similar intensity in both strain backgrounds (S7A Fig). Quantification of expression levels by qRT-PCR confirmed the result of the Northern Blot (S7B Fig). We also performed qRT-PCR of several other targets (*CYC8*, endogenously expressed *MOT3*, *CBK1* and *SSL2*) and could also not detect any difference between wild type and *rrp6*Δ strain (S7C Fig). These results show that the nuclear exosome is not involved in the degradation of the short transcripts.

The presence of short transcripts could also be an indication for the involvement of termination factors. Moreover, similar experiments in *E*. *coli* clearly show that the RAP induced negative effect on elongation is mediated via the termination factor Rho [23]. In *S*. *cerevisiae*, two major termination pathways have been identified, the cleavage and polyadenylation factor (CPF)-cleavage factor (CF) dependent pathway being responsible for mRNAs, and the NNS (Nrd1-Nab31-Sen1) pathway, which is tightly connected to exosome function and necessary for termination of snoRNAs, cryptic transcripts and other RNA species [13,42–44]. The major components of both pathways are essential for cell survival. Therefore, we used the well-established Anchor-away technique to remove Nrd1 (NNS) and Rat1 (CPF-CF) from the nucleus. Nrd1-FRB and Rat1-FRB fusion proteins form a complex with the ribosomal subunit Rpl12A fused to FKBP12 in the presence of rapamycin. The assembled ribosomal large subunit is then quickly exported to the cytoplasm which leads to rapid reduction of the tethered nuclear Rat1 or Nrd1 proteins in the nucleus [13,45]. We treated strains Nrd1-FRB and Rat1-FRB with rapamycin for 1 hour or left them untreated and determined expression levels as already described in Fig 3. Anchoring of neither Nrd1 nor Rat1 affected the RAP effect of *MOT3*, *CYC8*, *CBK1* or *SSL2* (S7D and S7E Fig). Thus, these two termination factors do not play a role in down-regulation of transcript levels of RAP host genes.

The down-regulating RAP effect could also be a consequence of pausing and backtracking of RNA Pol II. The major element facilitating the recovery of paused RNA Pol II is the elongation factor TFIIS, encoded by the *DST1* gene in *S*. *cerevisiae* [5,26,46]. We speculated that deletion of *DST1* might lead to changes in expression patterns of RAP-containing genes and compared transcript levels of *MOT3*, *CYC8*, *CBK1* and *SSL1* in wild type and *dst1*Δ cells. We could not detect any significant changes of the RAP effect between wild type and mutant. (S7F Fig). These results show that RAP-derived short transcripts are not degraded via the NNS-exosome pathway and therefore, represent a species that is different from CUTs. Moreover, the (CPF)-cleavage factor (CF) dependent pathway is very unlikely to be involved in termination of the short transcripts. Finally, the elongation factor TFIIS has no impact on the RAP effect, indicating that recovery of paused RNA Pol II mediated by this factor does not play a role.

### RAP-induced intragenic termination in *cis* is a general phenomenon

**Fig 5:**
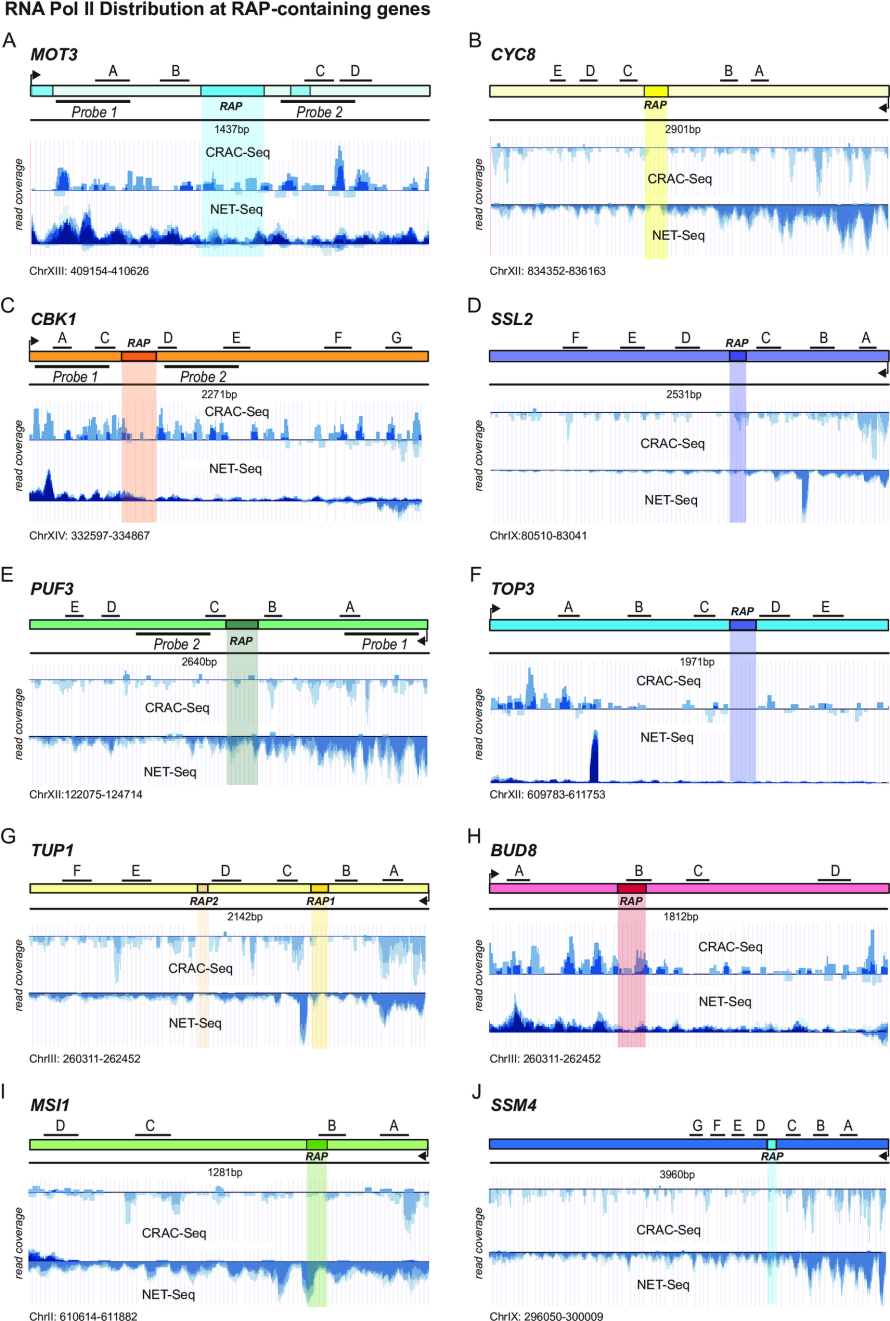
RNA Pol II distribution at RAP containing target genes. (A) to (J) Browser screenshots of CRAC-Seq (upper panels) and NET-Seq (lower panels) data sets for the targets analyzed in Figs 3 and S8. Independent overlapping data sets are indicated in shades of blue. RAP positions, amplicon positions for qRT-PCR (Fig 3) and positions of Northern Blot probes (Fig 4) are shown.

The fact that RAPs are directly binding to RNA Pol II in low nanomolar range prompted us to analyze the distribution of the protein complex at RAP-containing host genes. We speculated that RAPs might lead to pausing and/or premature termination of RNA Pol II in close proximity to the site of RAP transcription. To test this hypothesis, we performed Chromatin Immunoprecipitation (ChIP) of the largest RNA Pol II subunit Rpb1 at the selected targets analyzed for transcriptional effects in Fig 3. Some of the analyzed loci (*MOT3*, *CYC8*, *CBK1*, *SSL2*, *PUF3*, *MSI1*) show a slightly decreased occupancy of RNA Pol II downstream of the RAP (S8A–S8E Fig). The host genes *MOT3* and *SSM4* display small RNA Pol II peaks immediately upstream of the RAP which could be an indication for pause sites (S8A and S8J Fig). However, the *TOP3*, *TUP1* and *BUD8* loci show an even distribution of RNA Pol II all over the gene body (S8F, S8G and S8H Fig). These results could be explained by detection limits of the ChIP assay which is restricted to a low resolution of 200-300bp. Moreover, the obtained signals are not strand specific and can be distorted by antisense transcription at the 3’ end of genes ([47] and Fig 5C, 5H and 5I).

Therefore, we took advantage of the available NET-Seq and CRAC-Seq datasets which display base pair resolution and strand specificity [26,47]. In addition, these approaches do not disturb cellular functions by addition of crosslinking agents such as formaldehyde. Fig 5 shows browser screen shots (overlapping replicates shown in shades of blue) of the targets analyzed by ChIP in S8 Fig. In general, we could detect a drop of RNA Pol II occupancy downstream of the RAPs (especially pronounced in *CYC8*, *CBK1*, *SSL2*, *PUF3*, *BUD8*, *SSM4*) (Fig 5B, 5C, 5D, 5E, 5H and 5J). However, we could not observe any significantly pronounced RNA Pol II pause sites in close proximity to the RAPs. Thus, some RAPs might indeed cause premature termination within the gene body.

**Fig 6:**
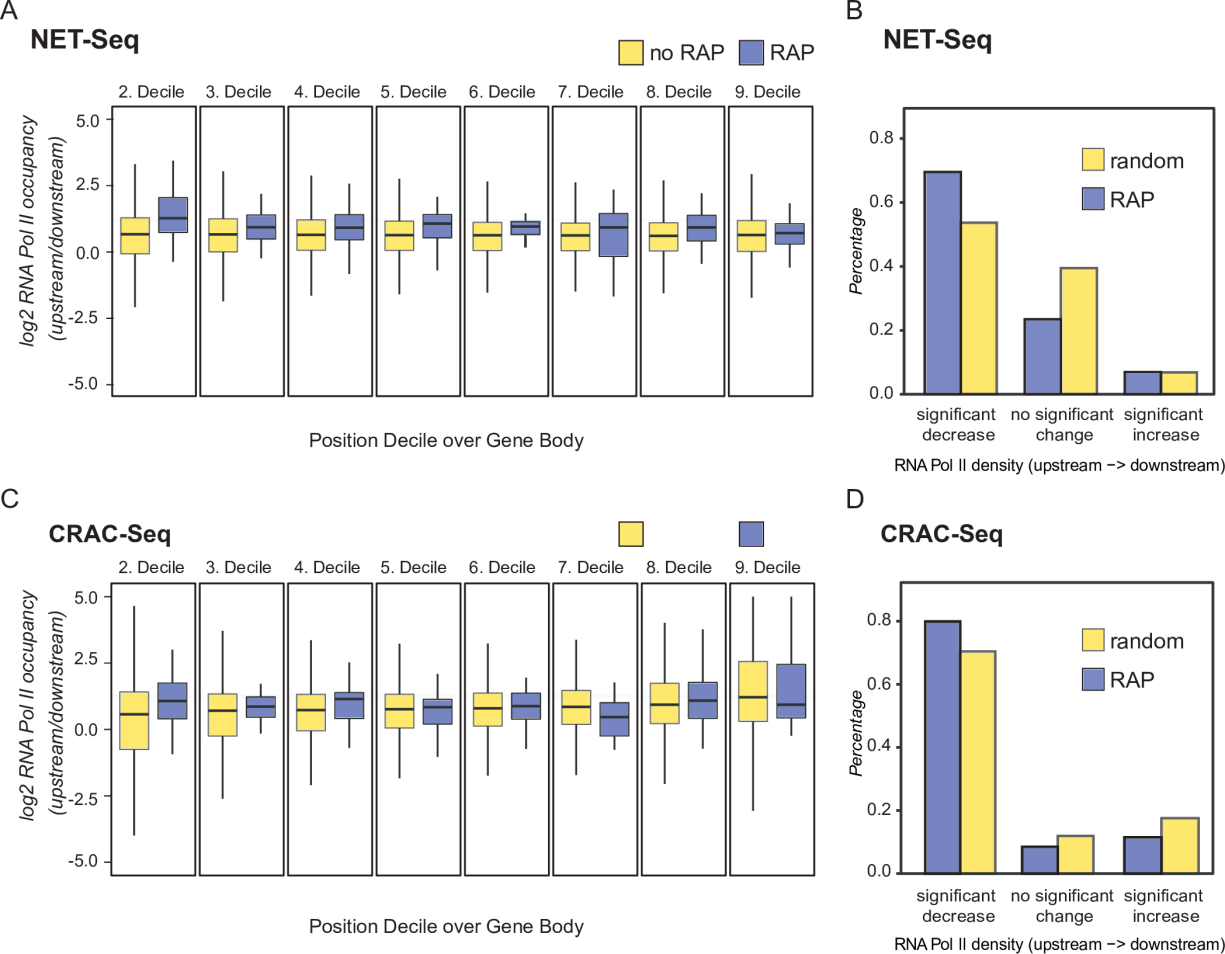
RAP host genes display intragenic termination. (A) and (C) show a gene centered view on RNA Pol II occupancy at RAP-containing genes and genes without RAPs determined by available NET-Seq (A) and CRAC-Seq (C) data sets. To avoid signal distortion by the RNA Pol II 5’ peak, the first 200nts (and for symmetry reasons also the last 200nts) were excluded from the analysis. The remaining gene body was binned into ten equally spaced segments (deciles) and the upstream/downstream ratio of RNA Pol II occupancy was calculated for each segment. Distribution of ratios from segments harboring a RAP are separately visualized (blue bars) and show a consistently higher upstream RNA Pol II density. (B) and (D) Ratio of RNA Pol II occupancy between genic regions upstream and downstream of RAPs tested by NET-seq (B) and CRAC-seq (D). For each gene position upstream and downstream of a RAP the number of reads per position from each experiment was deduced and tested for a significant increase, decrease, or no significant change between the upstream and downstream region coverage (Poisson test; p-value threshold < 0.01). To estimate an expected background distribution accounting for the general decrease of Pol II occupancy along the gene body, the analysis was repeated with 100 randomized positions, conserving the relative RAP positions within the harboring gene. Both data sets, NET-Seq (B) and CRAC-Seq (D), show that RAP containing genes more often display a significant decrease downstream of the RAP compared to the randomized background.

To confirm this assumption on a global scale we used two different strategies. In a first approach we removed the first 200nts of every gene to avoid signal distortion caused by RNA Pol II accumulation immediately downstream of the TSS ([26,48] and S9A and S9B Fig). Gene bodies of RAP-containing loci and genes without RAPs were split up into ten equally spaced segments. For each segment the mean signal strength ratio between all upstream and all downstream regions was calculated. This approach shows that ratios deduced by this way are systematically shifted to values greater than 1, which corresponds to a general decrease of RNA Pol II occupancy towards the 3’ end of the genes (S9A and S9B Fig). This pattern has been described in several previous publications [26,47,48]. However, and importantly, genes harboring a RAP, display consistently higher ratios between all upstream and downstream regions (Fig 6A and 6C). Therefore, RAP host genes (blue bars) have higher levels of RNA Pol II towards the 5’ end compared to genes without RAPs (yellow bars).

In a second, RAP focused approach the ratios of RNA Pol II occupancy densities upstream (gene start to RAP) and downstream (RAP to gene end) were calculated and tested for significance. As a background control we used randomized positions preserving the relative location of the RAPs within the genes. The difference between expected frequencies from the control and the observed counts of the RAP containing genes was tested for significance by using a chi-squared test (p-values of 3e-53 and 6e-09 for NET-Seq and CRAC-seq data, respectively). We observed that RAP host genes have a higher tendency for a decrease of RNA Pol II density compared to the randomized control (Fig 6B and 6D). These observations support that RAPs can lead to depletion of RNA Pol II levels in course of elongation.

## Discussion

A key step in evolution is the shift of RNA-driven RNA synthesis to protein-catalysed RNA synthesis. For this to occur RNAs and proteins had to co-evolve accompanied by a continuous cross-talk between the two macromolecules. We hypothesized that during protein-catalysed RNA synthesis the nascent RNA constantly interacts with RNA polymerases leading to elongation kinetics that are dependent on the underlying RNA sequence. In order to deepen our understanding of the cross-talk between RNA polymerases and nascent RNA, we searched for RNA sequences that have the capacity to interact with the transcription machinery. Previously, we identified such RNA sequences (termed RAPs) in a genomic SELEX experiment using the *E*. *coli* genome as source of RNAs and bacterial RNA Pol as bait [23]. For the study presented here, we performed a similar approach with a library derived from the yeast genome and yeast RNA Pol II as a bait. Genomic SELEX is an efficient method to identify RNA domains (sequence and/or structure) that exert high affinity to a chosen bait independently on expression levels of the transcripts. Even RNA sequences that are lowly or virtually not expressed in the cell, like the ARIA strand of telomeres, can be analysed.

Similar to the approach performed in *E*. *coli*, the primary outcome of the SELEX experiment is that the obtained sequences do not manifest a single common motif but are rather diverse. RNA Pol II is a very large protein complex composed of 12 subunits, each of them containing potential interaction sites for RNA and thus, the diversity of the identified sequences is not utterly surprising. Nevertheless, we could identify some motifs such as the CAA-rich motif in a subset of characterized aptamers. The tested RAP-containing genes harbouring this sequence motif all showed a drop of transcript levels downstream of the RAP (*CYC8*, *CBK1*, *PUF3*). Assuming that RAPs with diverse sequences could interfere with transcription we tested mRNA levels flanking the aptamers of 14 RAP-containing genes in their endogenous context. We found that 10 of them had diminished transcript levels downstream of the RAPs. RAPs that were found to be inactive in our approach could still have an impact on transcription that requires detection via different assays. Trans-acting regulatory RNAs such as the B2 or Alu RNAs or the synthetic Fc aptamer that binds yeast RNA Pol II have been previously identified and some RAPs might work in a similar manner [15,18,49–52]. In this study we focused on *cis*-acting RAPs and did not test potential effects in *trans*. RAPs could also positively affect transcription via a mechanism that is similar to the antiterminating effect of the *E*. *coli* phage *putR* and *putL* elements [21]. Since many mRNA processing events in eukaryotes take place co-transcriptionally, intragenic RAPs might interfere with many of these processes.

Interestingly, we found that RAPs encoded in subtelomeric and telomeric regions also have a repressive effect on elongation. This indicates that cells might use RAPs for silencing of certain regions such as telomeres, origins of replication or centromeres. Telomere ends are covered with the DNA-binding protein Rap1 with a recognition sequence that is encoded in both strands (5’-ACACC(n)_1-3_ACACC-3’) (53). The number of Rap1 molecules associated with the telomere is the major determinant of telomere length and Rap1 recruits the silencers Sir2, Sir3 and Sir4 [33]. Moreover, Rap1 has been shown to be associated with DNA throughout the genome working as both, transcriptional activator and repressor [53,54]. However, we propose that the repressive effect of telomeric RAPs is not a simple consequence of Rap1 binding because the GU-rich control sequence does not have any effect on elongation. Therefore, we suggest that the observed RAP effect is indeed caused by the telomeric CA-rich transcript.

For RAP sequences containing the CA-rich motif (and the CAA-rich motif of *CYC8* and *CBK1*) we could confirm that the underlying double stranded DNA or encoded protein sequence are not responsible for the RAP effect (see Fig 2). Thus, RAPs do not just represent simple DNA-encoded obstacles for the elongating RNA Pol II. However, at the moment we cannot exclude that interaction of protein factors with the single stranded non-template DNA strand is the cause for the down-regulation of transcription. However, we do not think that this scenario is very likely, since RAPs were selected for direct interaction with RNA Pol II *in vitro* in the absence of additional protein factors during the SELEX procedure.

Elongation of mRNA is not a homogenous process. Several factors influence the elongation rate of individual genes. In fact, the dynamics of elongating RNA Pol II are highly gene-specific suggesting that the underlying DNA sequence and the regulatory features of a gene are significant factors for elongation efficiency [1–4,6,48]. RNA polymerase II faces several barriers such as the nucleosome dyad and has been shown to undergo pausing throughout the transcribed region [26,47]. We hypothesized that RAPs might be a cause for pausing of elongating RNA Pol II at the site of their expression and some of our analyzed RAP host genes show small peaks of RNA Pol II in our ChIP experiments (S8 Fig, *MOT3*, *PUF3*, *SSM4*) which could indeed point to backtracked elongation complexes. However, available high-resolution NET-Seq and CRAC-Seq data sets show that RNA Pol II occupancy levels are slightly but significantly diminished downstream of RAP sequences and we could not observe any RNA Pol II peaks on single loci in these setups. This finding rather points to a RAP-induced intragenic termination event which is corroborated by the fact that TFIIS does not have any impact on the upstream/downstream transcript ratios at RAP host genes. Nevertheless, we cannot completely exclude the possibility that pausing and backtracking might be involved since we only analyzed a small number of RAP-containing genes and pausing events might simply be masked in our genome-wide analysis. Moreover, we have not investigated the impact of post-transcriptional events, such as RNA degradation in the cytoplasm, in this study.

Interestingly, the observed diminished RNA Pol II levels seem not to be mediated by the two major termination pathways in yeast. This is in contrast to the findings in *E*. *coli* where the inhibitory RAP effect is clearly dependent on the activity of the termination factor Rho. Therefore, we speculate that *cis*-acting RAPs in *S*. *cerevisiae* might exert their effects by directly regulating elongating RNA Pol II without the need of additional protein factors.

The disruption of elongation at RAP host genes resulted in the generation of multiple short transcript species with undefined 3’ ends identified by Northern Blotting and 3’ RACE. Although some of these transcripts end within or in close proximity to the RAPs, the majority ends upstream which could be explained by degradation events. A recent publication proposed a model where the early elongation complex is always in a state poised for termination leading to short, unstable transcripts that are substrates for the NNS-Exosome pathway similar to CUTs [47]. The majority of these so-called S CUTs (short, promoter-proximal, sense oriented ncRNAs) ends in a window of 200bp downstream of the TSS. However, the short transcripts observed at RAP host genes by Northern Blot and 3’ RACE are definitely longer, are not degraded by the nuclear exosome and their expression levels do not change in the absence of the NNS-pathway. Thus, RAP-derived short transcripts are not similar to S CUTs but most probably represent a different species of ncRNAs. The question whether they have a relevant cellular function or are just by-products of transcriptional downregulation is a challenging subject for future approaches.

In conclusion, RAP-mediated control of transcription elongation is a phenomenon that is manifested by the cross-talk of specific sequences within the nascent RNA interacting with the elongating RNA polymerase. This phenomenon is not restricted to yeast RNA Pol II, as we also observed similar activities with *E*. *coli* RAPs and bacterial RNA polymerase [23]. RAPs identified in *E*. *coli* are able to modulate gene expression under stress conditions and strongly reduce transcription interference from antisense transcripts [23]. Therefore, the interaction of RAPs with RNA polymerases represents an important level of regulation that has a strong impact on the sequence composition of genomes because it provides RNAs with an intrinsic capacity to regulate their own expression.

## Materials and methods

### Strains and growth conditions

Strains used in this study are listed in S3 Table. For analysis of endogenous transcript levels *S*. *cerevisiae* strains were grown in YPD (2% Glucose) to early exponential growth phase (OD_600_ 0.8–1) and harvested by centrifugation followed by freezing in liquid nitrogen. Strains with RAP-containing transcripts under the control of the *GAL-10* promoter were grown in 2% raffinose to early exponential phase followed by addition of 2% galactose and harvest of cells after 10, 30 and 60 minutes. In case of the essential *CBK1* locus cells were grown in the presence of 2% galactose followed by addition of 2% glucose. Cells containing the endogenously expressed reporter construct were also grown in medium with raffinose followed by addition of galactose. Alternatively, reporter cells were exclusively grown in medium with 2% galactose. Anchor Away strains Nrd1-Frb-KanMX6 and Rat1-Frb-KanMX6 were grown to early exponential phase and treated with 1μg/ml rapamycin (LC-Laboratories) for 60 minutes or left untreated.

### SELEX procedure

The genomic library was created as described in [24] with genomic DNA purchased from Sigma as template. For genomic SELEX, fragments of 30–400 bp were selected by separating the DNA pool in a 10% denaturing gel and excising the desired size range. After transcribing the genomic library into RNA, the RNA pool was bound to the RNA Pol II of *S*. *cerevisiae* in an *in vitro* binding reaction as described in [24]. For the 6 cycles, RNA was added at 1μM and protein at 100nM. The binding buffer contained 10 mM HEPES pH 7.25, 40 mM NH_4_SO_4_, 10 μM ZnCl_2_, 1 mM KCl, 10 mM DTT, 5% glycerol and 10 mM MgCl_2_.

The DNA library from SELEX cycle 6 amplified by 16 cycles of PCR with Pfu polymerase (New England Biolabs) using primers with fixed part and added barcode for multiplexing according to [55] (fixFW_multi: GGAATTCGGAGCGGGACTGG, fixRV_multi: CGGGATCCTCGGGGCTGACTTG (Sigma)). Library preparation (adapter ligation step) and deep sequencing were performed at the VBCF NGS Unit (www.vbcf.ac.at). The SELEX library was sequenced using a GAIIx with paired-end 76 base-pair reads. 3’ adaptors were removed using cutadapt (doi: 10.14806/ej.17.1.200) and 5’ sequencing primers and barcodes were removed with a custom script. Read pairs of which either read was less than 18nt long were discarded. After primer removal and demultiplexing, paired-end reads were aligned to the sacCer2 genome as downloaded from the UCSC genome browser (doi:10.1101/gr.229102) using bowtie2 (doi:10.1038/nmeth.1923) using the settings–score-min G,14,8 -N 1–reorder -p 6–dovetail–local. Mapped mate pairs were filtered such that the distance between 5’ ends were no less than 20 and no more than 500 bases apart on the same chromosome.

RAP peaks were then determined using a custom peak finding algorithm. Mate pairs were counted between the 5’ ends of the mapped insert. Regions containing 5 or more reads were then smoothed using a Gaussian kernel with a window size of 5. The first derivative was estimated as the difference between the smoothed signal current and following position. Peaks were detected by selecting pairs of maximal ascending and descending positions which were between 20 and 500 bases long.

### Creation of the reporter system

The RAP-reporter strain was created by sequential homologous recombination into the region between the *MAL11* and the *MAL13* locus on chromosome VII (Chr VII:1072353–1072472). First, natMX::*GAL-10prGFP* was amplified by PCR using primers with homologous overhangs for genomic integration (S4 Table). Plasmid pYM-N25 (Euroscarf) was used as a template. After transformation selected clones were checked for correct integration by PCR and fluorescence microscopy detecting the GFP signal. Next, the *HIS3* open reading frame was amplified from plasmid pRS313 and integrated downstream of *GFP* thereby excluding the GFP STOP codon. Clones were selected for growth on medium lacking histidine and control PCR. Finally, the *URA3* ORF including the STOP codon was amplified from plasmid pRS316 and integrated between *GFP* and *HIS3*. Cells were selected for growth on medium lacking uracil and death on medium lacking histidine. The resulting strain could be used for integration of several RAP and control sequences: The *URA3* ORF including the STOP codon was replaced by the corresponding sequence and cells could be selected by growth on SC-HIS + Gal, SC +5-FOA +Gal and lethality on SC-URA. The inserted RAP and control sequences were designed with flanking regions (~100bp) overlapping to *GFP* (5’ end) and *HIS3* (3’ end) and submitted for synthesis to Genewiz®. All strains were controlled for integration by PCR. Control primers are indicated in S4 Table.

### RNA extraction and quantitative RT-PCR

Cells were grown to early exponential phase and harvested by centrifugation. RNA was isolated using Phenol-Chloroform extraction essentially as described before [56,57]. Concentration of the RNA was determined using Nanodrop 2000 (ThermoScientific) and 20μg were used for DNAseI digestion (Roche). RNA was purified by Phenol-Chloroform extraction followed by a second round of DNaseI digestion. Reverse Transcription of 2μg of DNase-free RNA was performed using Protoscript II RT (New England Biolabs) at 42°C for 60 minutes. The cDNA was diluted 1:10 and transcript levels were determined in a total volume of 25μl by quantitative Real-Time PCR (Eppendorf Mastercycler) relative to the constitutively expressed *IPP1* transcript [57]. Data were normalized to the first amplicon at the 5’end. Primer positions can be found in the corresponding figure and S4 Table. All experiments were repeated at least three times.

### Northern Blotting

Northern Blotting was performed as described before [56]. Purified total RNA was separated on a 1% agarose gel containing 1% formaldehyde. RNA was blotted on nitrocellulose membrane (GE healthcare) using 20x SSC as a transfer buffer. Probes were amplified from genomic DNA and labeled with alpha dATP using Stratagene® labeling kit II. Probes were hybridized at ~65°C over night. Membranes were washed 2x at room temperature and 2x at 65°C and exposed to phosphoimager for 4 hours up to 24 hours.

### Chromatin Immunoprecipitation

ChIP was performed as described [56]. Cells were grown to OD~0,800–1.000 followed by crosslinking (1% formaldehyde) and washing with 1xTBS. Cell pellets were stored at -20°C. The pellets were re-suspended in 600μl of Lysis buffer (1%Triton, 1mg/ml Sodium deoxycholate, 50mM Hepes, 140mM NaCl, 1mM EDTA, Benzamidine, PMSF), disrupted by glass beads and chromatin was fragmented to an average size of 200bp using ultrasonic sound. Dynabeads Protein G and antibody against Rpb1 (8WG16, Covance Inc.) were used for immunoprecipiation. Crosslinking was reversed by incubation at 65°C over night and ChIP DNA was purified by Phenol-Chloroform extraction. 5μl of DNA were quantified by qPCR (total volume 25μl). Primer sequences can be found in S4 Table. At least 3 biological replicates were analyzed.

### 3’ RACE

6μg of DNA-free total RNA were used for dephosphorylation by T4PNK (NEB®) in Buffer: 400mM Tris-HCl pH6.5, 400mM MgAc, 20mM Mercaptoethanole. Dephosphorylated, purified RNA was phosphorylated by Calf intestinal phosphatase (NEB®). 5pmol of 3’ adaptor (AAUGGACUCGUAUCACACCCGACAA, Thermo Scientific®) were phosphorylated using T4PNK (NEB®). After extraction by Phenol-Chloroform RNA ligation was performed at 17°C over night using T4 RNA ligase (NEB®). The ligation reaction was purified by PCl-extraction and cDNA was synthesized using random nonamers and Protoscript RT II (NEB®). For amplification target-specific forward primers and an adapter-specific reverse primer were used. PCR-products were cloned into pGEM-T Easy vector (Promega®) and sequenced.

### Data analysis

#### Expression analysis

For the correlation of transcript abundance of RAP hosting genes the mRNA-seq data set from Churchman et al. was used (SRA accession numbers SRR072819, SRR072820, SRR072821, SRR072822) [26]. Read quality was controlled using FastQC, adapter and low-quality reads were removed using BBduk from the BBmap software suite (sourceforge.net/projects/bbmap/). Reads were mapped using segemehl version 2.0 [58]. Multimapper and PCR duplicates were removed from further analysis. Read end position were accumulated using bedtools multicov [59]. TPM-values (Transcripts Per Million values) were calculated with scripts from the ViennaNGS package [60]. Genes were sorted according to their TPM values and visualized in the form of a cumulative distribution plot. Deviations from the uniform distribution were evaluated for manually chosen segments using poisson.test function from R (https://www.r-project.org/).

#### GO terms

GO term enrichment analysis for all protein coding genes containing at least one RAP in contrast to the remaining yeast genes was performed using GOrilla [61]. Enrichment of RAP locations with respect to their genomic context was based on the genome annotation retrieved from Saccharomyces Genome Database (SGD, http://www.yeastgenome.org) and SUTs& CUTs [36]. Overlap with RAP coordinates and genome annotation was performed with bedtools intersect. Overlap of one base was enough to assign a RAP to a genomic feature. Since different feature classes and RAPs have different sizes the expected values for RAP within a certain feature class is not straight forward derivable. Therefore, we empirically deduced the expected values and the associated p-values simulating 100,000 bootstrapped experiments with randomized RAP location, preserving the respective RAP and feature lengths.

#### Motif search

To uncover putative enriched sequence motifs associated with RAPs the Homer software suite was applied [27]. An important issue of sequence motif enrichment analysis is the choice of the correct sequence background. Therefore, we tested different subsets of annotated RAPs, defined by their genomic context, individually. As background for each analysis a 100-fold sampled sequence set was used, featured with the same length distribution and, since sampled from the same genomic context, a representative nucleotide composition. Categories used were the following: long terminal repeat, ncRNA, ORF, rRNA, snoRNA, telomere, telomeric repeat, tRNA, X element combinatorial repeats, X element core sequence, Y' element, gene cassette, mating locus, ncRNA, ORF, pseudogene, retrotransposon, rRNA, telomere, telomeric repeat, transposable element gene, X element combinatorial repeats, X element core sequence, Y' element, ARS, and all RAPs.

#### RNA Pol II occupancy

For the analysis of RNA Pol II occupancy NET-seq data from Churchman et al. (SRA accession numbers SRR072814, SRR072815, SRR072816, SRR072817, SRR072818) and CRAC-seq data from Milligan et al. (SRA accession numbers SRR2056997 and SRR2056998) were used [26,47]. Read quality was controlled using FastQC, adapter and low quality reads were removed using BBduk from the BBmap software suite (sourceforge.net/projects/bbmap/). Reads were mapped using segemehl version 2.0 [58]. Read ends were quantified with in-house scripts, multi-mapper signals were proportionally distributed to all mapping sites. To test the overall behavior of RNA Pol II occupancy along the gene body, each gene was split into 10 equally spaced segments, omitting the first 200nts to avoid signals from the transcription initiation region. For each segment the relative change of RNA Pol II occupancy in the form of read ends per genomic position between all upstream and downstream segments was deduced. The log2-ratio distribution over all genes was visualized and compared between segments with and without a RAP. Positive log2-ratios correspond to an RNA Pol II enrichment in the upstream segments.

To analyze the effect of RAPs onto RNA Pol II occupancy from a RAP perspective, the ratios of mean RNA Pol II occupancy upstream and downstream of each RAP from within annotated genes were evaluated based on a Poisson test if significantly different from 1 (p-value <0.01). If not, the ratio was set to 0. To obtain a background distribution each RAP was randomly moved to 100 genes preserving its position relative to the gene boundaries. The analysis was repeated with this randomized background data set. The distributions of log2 ratios between the upstream and downstream gene body were tested for equality with a nonparametric Kolmogorov-Smirnov test.

## Supporting information

S1 Fig (A) Class distribution of RAPs. Analysis of the occurrence of RAPs in different genomic contexts. For each category the number of RAPs overlapping at least one base with any annotated feature was counted. Since the feature and the RAPs differ in length the expected count was calculated by randomizing the RAP location preserving its length, and the analysis was re-done 100,000 times. The lower panels show the log2-transformed enrichment of observed counts/expected counts. Categories are marked according to the empirically determined p-value, <*> and <**> represent significance levels p-value <0.01 and <0.001, respectively. (B) and (C) Gene Ontology enrichment analysis for all protein coding genes harboring a RAP. All terms and their relation from the domain of biological process with a p-value below 10^(-6) are depicted. The RAP containing gene set is highly enriched in genes associated with regulatory function, especially transcription regulation via RNA polymerase II.
(PDF)

S2 Fig A reporter system for analysis of RAP effects. (A) The reporter system for detection RAP effects was created by sequential homologous recombination at a non-transcribed region between *MAL11* and *MAL13* on chromosome VII (Chr VII:1072353–1072472). After integration of natMX::GAL1prGFP (amplified from plasmid pYMN-25), a HIS3 cassette was integrated downstream of the GFP followed by recombination of a URA3STOP cassette enabling selection for growth on medium lacking uracil. The URA3STOP cassette could be exchanged for any RAP or control sequence. Resulting transformants can be selected for growth on medium lacking histidine or containing 5-FOA. (B) Location of primer pairs for qRT-PCR. (C) Expression profile of the *GFPHIS3* reporter during growth in medium with 2% galactose as a sole carbon source. Expression levels are equally distributed over the fusion transcript. (D) The *GFPHIS3* fusion transcript can be induced via the *GAL1-10* promoter. Cells were grown in 2% raffinose to early exponential phase and galactose was added to a final concentration of 2%. Aliquots were taken at indicated time points. (E) Effect of *CYC8*-RAP in the reporter construct. Cells containing the reporter construct with (GFPRAPHIS3) or without (GFPHIS3) the full-length RAP of the *CYC8* locus (see S3 and S5 Tables) were grown on 2% raffinose to exponential growth phase and the *GAL1-10* promoter was induced by addition of 2% galactose. Aliquots were taken after 10, 30 and 60 minutes and expression levels were determined by qRT-PCR after reverse transcription using random nonamers (see Fig 2 and materials and methods). Values were normalized to GFP levels (Primer pair B).
(PDF)

S3 Fig Transcriptional landscape of selected RAP-containing target genes in wild type and *rrp6*Δ. Browser pictures resulted from high resolution tiling microarrays (8bp) taken from the data set by Xu et al. (36). cDNA synthesis was performed using a combination of oligodT and random primers in this setup. Signal intensities for both DNA strands (W and C) for the different profiled samples are shown on the y-axis. Three independent results from BY wild type and *rrp6*Δ strains are shown in parallel. No antisense transcripts can be observed at the sites of the analyzed RAP-containing genes.
(PDF)

S4 Fig (A) to (F) Transcript levels of 6 selected target genes (*ACT1*, *PDA1*, *RPN2*, *PGK1*, *STE5 and CET1*) not containing RAPs. Reverse transcription was performed with random nonamers and expression levels were quantified by qRT-PCR using amplicons as indicated in the respective figures. Values were normalized to the first 5’ amplicon. (G) to (I) RAP-containing genes *PRY3*, *SRV2* and *TUB4* do not show any drop of transcript levels downstream of the RAPs.
(PDF)

S5 Fig (A) Schematic of position of Northern Blot probes at the *GAL1* locus under the control of its endogenous *GAL1-10* promoter. (B) Northern Blot using the probes indicated in A).
(PDF)

S6 Fig (A) to (C) 3’RACE results for *MOT3*, *CBK1* and *PUF3* under the control of the inducible *GAL1-10* promoter. Positions of the RAPs and the used gene specific forward primers are indicated. PCR products resulting from amplification with the gene specific primer and the adapter specific primers are shown below the sequences. For *MOT3* the transcripts lengths range from ~200-1100bp with a forward primer starting at 186bp, the majority having a size of 500bp. For *PUF3* the lengths range from ~100-1000bp when forward primer 127fw is used and from 100–750 when primer 565fw is used. *CBK1* shows a size distribution of 100-500bp with primer 81fw. The PCR products were cloned into the p-GEM vector (Promega) and 7–11 clones were sent for sequencing to confirm specificity. The frequency of the identified 3’ends of these clones are indicated.
(PDF)

S7 Fig Termination and degradation of RAP-derived short transcripts is not dependent on the NNS-Exosome pathway. (A) Northern Blot as presented in Fig 3C but here also the nuclear exosome mutant *rrp6*Δ was analyzed. The short unstable transcripts detected with the upstream *probe1* are similarly expressed in wild type and mutant strain. (B) qRT-PCR analyzing the same samples used for Northern Blotting show similar expression patterns in wild type and *rrp6*Δ mutant. (C) to F) qRT-PCRs of *MOT3*, *CYC8*, *CBK1* and *SSL2* wild type and mutant cells. Reverse transcription of DNA-free RNA was performed using random nonamers and quantification was done using primers upstream and downstream (B and E for *MOT3*, D and G for *CYC8*, C and E for *CBK1*, B and E for *SSL2*) of the RAP similar as in Fig 3) Nuclear exosome mutant lacking the intranuclear subunit Rrp6. (D) Mutant of the NNS-pathway component Nrd1. Nrd1-FRB cells were treated with 1μg rapamycin for 1 hour or left untreated. (E) Mutant of polyA-dependent termination pathway endonuclease Rat1. Rat1-FRB cells were treated with 1μg rapamycin for 1 hour or left untreated. (F) Mutant lacking the elongation factor Dst1, a component of TFIIS.
(PDF)

S8 Fig RAP-containing genes have depleted levels of RNA Pol II downstream of RAP sequences. (A) to (J) Chromatin Immunoprecipitation of RNA Pol II (Rpb1) at RAP-containing target genes. Similar amplicons as for analysis of expression profiles were used (see Fig 2) for qRT-PCR. The majority of the analyzed loci show more RNA Pol II binding upstream of the RAP (except *BUD8* and *TUP1*). The *MOT3* and *SSM4* loci seem to have a pause site immediately upstream of the RAP.
(PDF)

S9 Fig (A) Relative RNA Pol II occupancy across the gene body as seen in the CRAC-seq and NET-seq data (26,47). All the positions in all the annotated genes are bin into ten deciles, so that the first 10% of the gene bases are grouped in the 1^st^ decile. The read signal in each decile is further normalized with the total read signal for each gene. Both data sets show that the average RNA Pol II occupancy is highest at the 5’ end of ORFs.
(PDF)

S1 Table Yeast RAPs identified in this study. (XLSX)

S2 Table RAP-containing target genes. (DOCX)

S3 Table Strains used in this study. (DOCX)

S4 Table Primers used in this study. (DOCX)

S5 Table Sequences tested in the reporter system. (DOCX)
