# Supplementary material for: Nascent RNA signaling to yeast RNA Pol II during transcription elongation
Source: PLoS One. 2018 Mar 23;13(3):e0194438. doi: 10.1371/journal.pone.0194438 (PMC5865726; doi:10.1371/journal.pone.0194438)
Supplement: S1 Fig — (A) Class distribution of RAPs. Analysis of the occurrence of RAPs in different genomic contexts. For each category the number of RAPs overlapping at least one base with any annotated feature was counted. Since the feature and the RAPs differ in length the expected count was calculated by randomizing the RAP location preserving its length, and the analysis was re-done 100,000 times. The lower panels show the log2-transformed enrichment of observed counts/expected counts. Categories are marked according to the empirically determined p-value, <*> and <**> represent significance levels p-value <0.01 and <0.001, respectively. (B) and (C) Gene Ontology enrichment analysis for all protein coding genes harboring a RAP. All terms and their relation from the domain of biological process with a p-value below 10^(-6) are depicted. The RAP containing gene set is highly enriched in genes associated with regulatory function, especially transcription regulation via RNA polymerase II. (PDF) [file pone.0194438.s001.pdf]

Supplementary Figure 1

A Class distribution

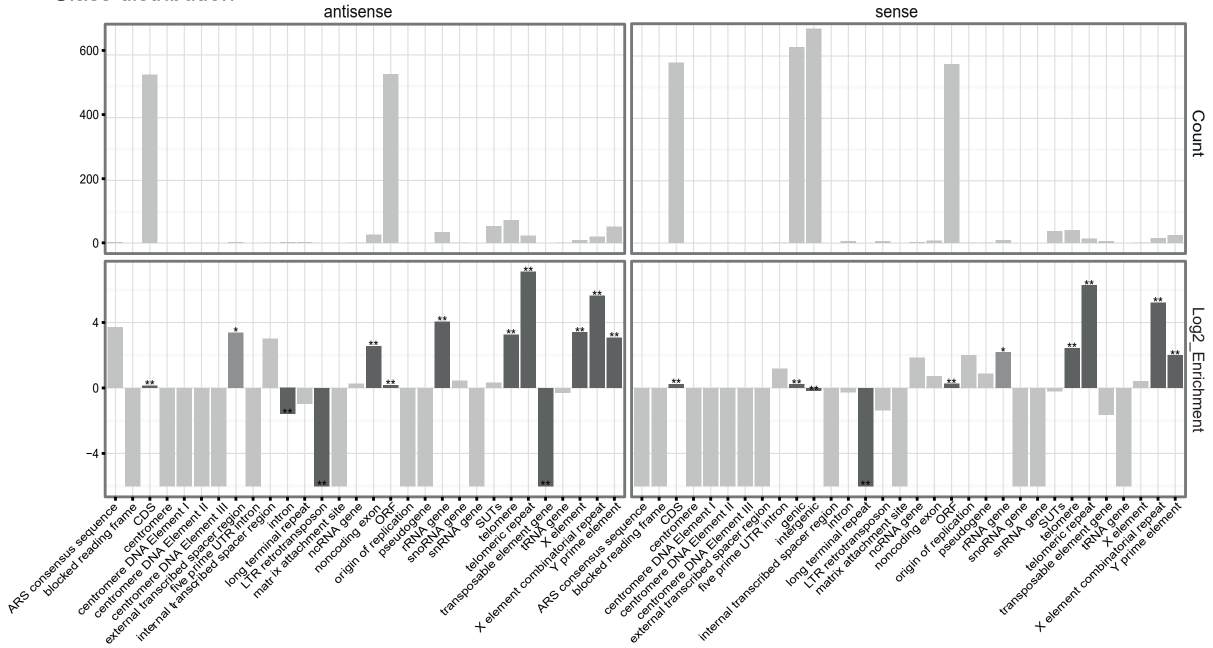

B GO Terms

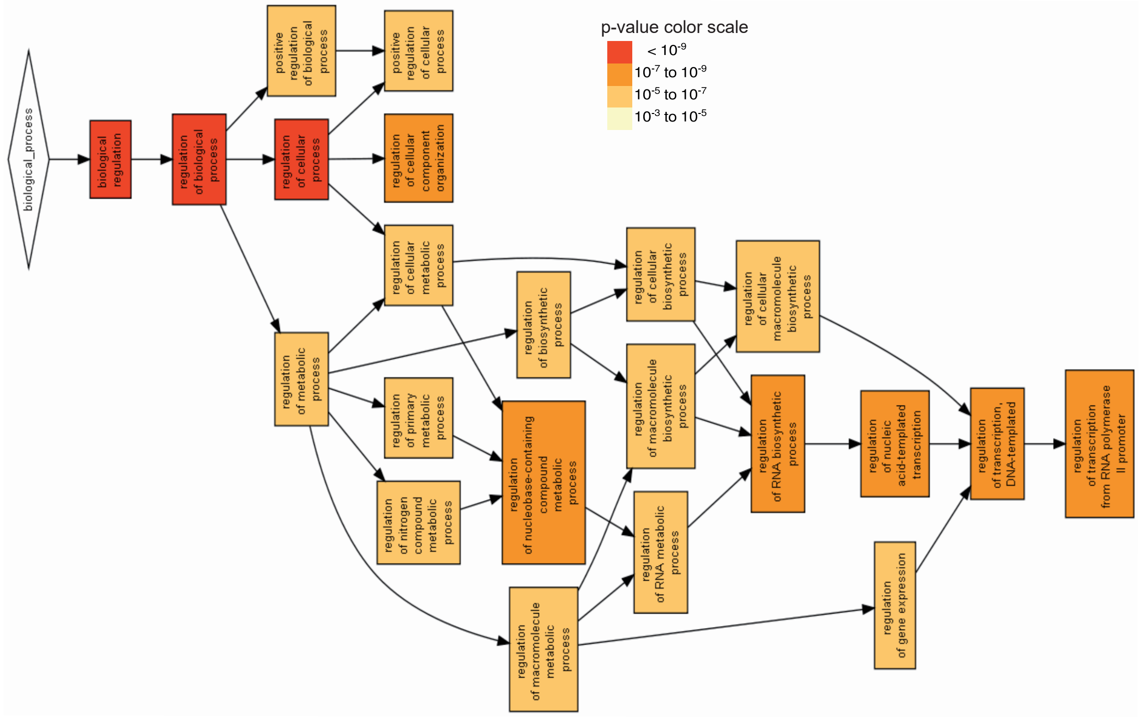

C

GO term description	p-values	FDR q-value	Enrichment	e)	f)
biological regulation	**1.44E-11**	7.31E-08	1.42	2043	224
regulation of cellular process	**2.33E-11**	5.93E-08	1.51	1590	185
regulation of biological process	**4.02E-11**	6.83E-08	1.48	1692	193
regulation of cellular component organization	**2.77E-09**	3.53E-06	2.04	438	69
**regulation of transcription from RNA polymerase II promoter**	**2.06E-08**	2.10E-05	1.95	459	69
**regulation of transcription, DNA-templated**	**4.26E-08**	3.61E-05	1.71	715	94
regulation of nucleobase-containing compound metabolic process	**5.06E-08**	3.68E-05	1.64	832	105
regulation of RNA biosynthetic process	**6.93E-08**	4.42E-05	1.69	722	94
regulation of nucleic acid-templated transcription	**6.93E-08**	3.92E-05	1.69	722	94
regulation of RNA metabolic process	**1.03E-07**	5.26E-05	1.66	759	97
regulation of gene expression	**1.32E-07**	6.11E-05	1.58	922	112
regulation of biosynthetic process	**1.40E-07**	5.94E-05	1.57	923	112
positive regulation of biological process	**1.67E-07**	6.54E-05	1.7	673	88
regulation of cellular macromolecule biosynthetic process	**1.86E-07**	6.77E-05	1.59	874	107
positive regulation of cellular process	**1.98E-07**	6.73E-05	1.7	655	86
regulation of macromolecule metabolic process	**2.24E-07**	7.12E-05	1.49	1140	131
regulation of cellular metabolic process	**2.25E-07**	6.76E-05	1.49	1129	130
regulation of macromolecule biosynthetic process	**2.26E-07**	6.41E-05	1.58	888	108
regulation of metabolic process	**2.31E-07**	6.20E-05	1.47	1208	137
regulation of cellular biosynthetic process	**2.50E-07**	6.36E-05	1.56	922	111
regulation of primary metabolic process	**2.55E-07**	6.18E-05	1.5	1109	128
regulation of nitrogen compound metabolic process	**8.00E-07**	0.000185	1.48	1075	123

e) Total number of genes associated with GO Term **non RAP**

f) Total number of genes associated with GO Term **RAP-containing (sense)**

total number of genes: 5853

total number of RAP-containing genes (sense): 451
