# Supplementary figures and images for: Nascent RNA signaling to yeast RNA Pol II during transcription elongation
Source: PLoS One. 2018 Mar 23;13(3):e0194438. doi: 10.1371/journal.pone.0194438 (PMC5865726; doi:10.1371/journal.pone.0194438)

Supplementary Figure 2

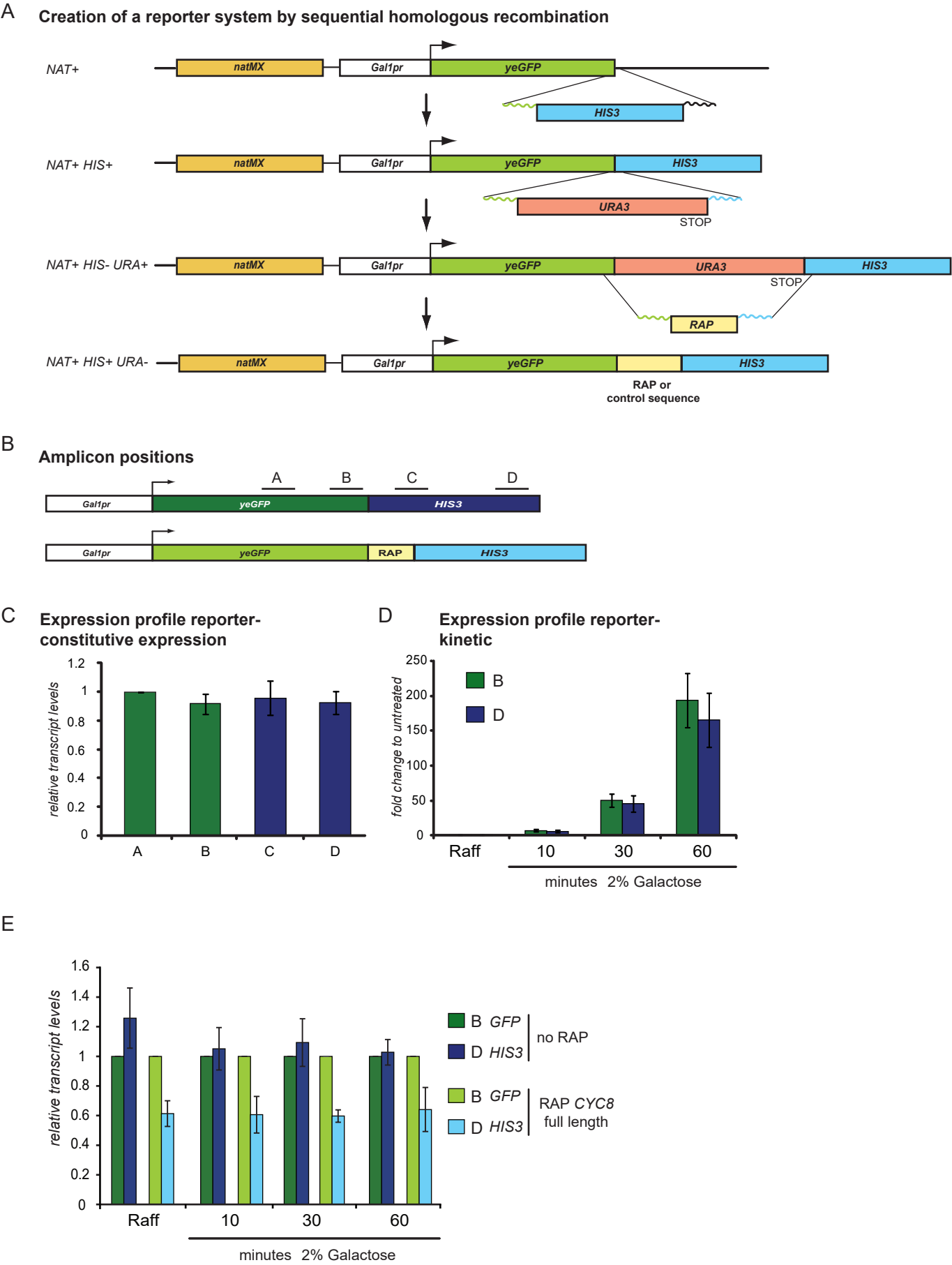

Supplement: S2 Fig — (A) The reporter system for detection RAP effects was created by sequential homologous recombination at a non-transcribed region between MAL11 and MAL13 on chromosome VII (Chr VII:1072353–1072472). After integration of natMX::GAL1prGFP (amplified from plasmid pYMN-25), a HIS3 cassette was integrated downstream of the GFP followed by recombination of a URA3STOP cassette enabling selection for growth on medium lacking uracil. The URA3STOP cassette could be exchanged for any RAP or control sequence. Resulting transformants can be selected for growth on medium lacking histidine or containing 5-FOA. (B) Location of primer pairs for qRT-PCR. (C) Expression profile of the GFPHIS3 reporter during growth in medium with 2% galactose as a sole carbon source. Expression levels are equally distributed over the fusion transcript. (D) The GFPHIS3 fusion transcript can be induced via the GAL1-10 promoter. Cells were grown in 2% raffinose to early exponential phase and galactose was added to a final concentration of 2%. Aliquots were taken at indicated time points. (E) Effect of CYC8-RAP in the reporter construct. Cells containing the reporter construct with (GFPRAPHIS3) or without (GFPHIS3) the full-length RAP of the CYC8 locus (see S3 and S5 Tables) were grown on 2% raffinose to exponential growth phase and the GAL1-10 promoter was induced by addition of 2% galactose. Aliquots were taken after 10, 30 and 60 minutes and expression levels were determined by qRT-PCR after reverse transcription using random nonamers (see Fig 2 and materials and methods). Values were normalized to GFP levels (Primer pair B). (PDF) [file pone.0194438.s002.pdf]

Supplementary Figure 3

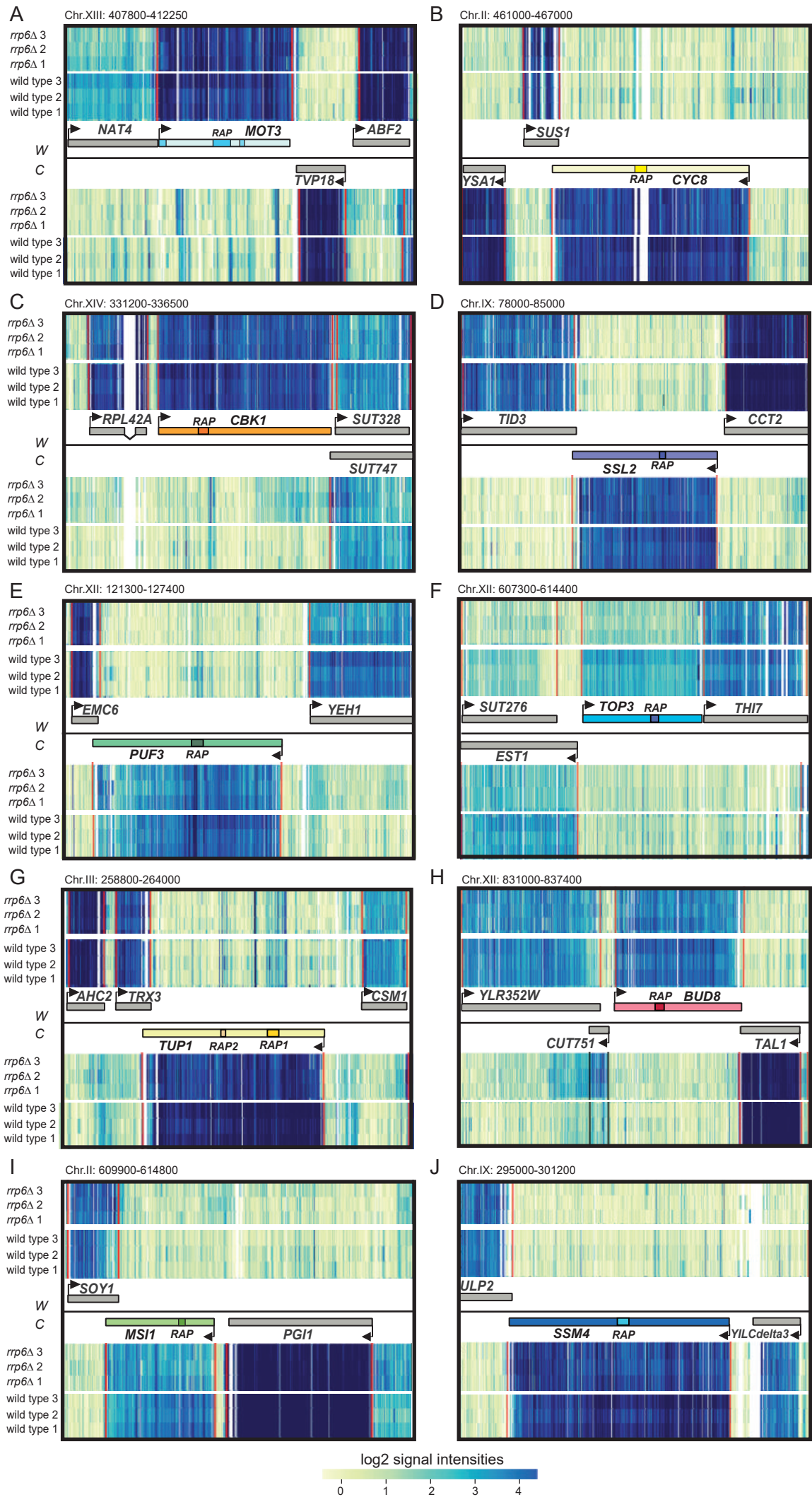

Supplement: S3 Fig — Browser pictures resulted from high resolution tiling microarrays (8bp) taken from the data set by Xu et al. (36). cDNA synthesis was performed using a combination of oligodT and random primers in this setup. Signal intensities for both DNA strands (W and C) for the different profiled samples are shown on the y-axis. Three independent results from BY wild type and rrp6Δ strains are shown in parallel. No antisense transcripts can be observed at the sites of the analyzed RAP-containing genes. (PDF) [file pone.0194438.s003.pdf]

Control genes without RAPs

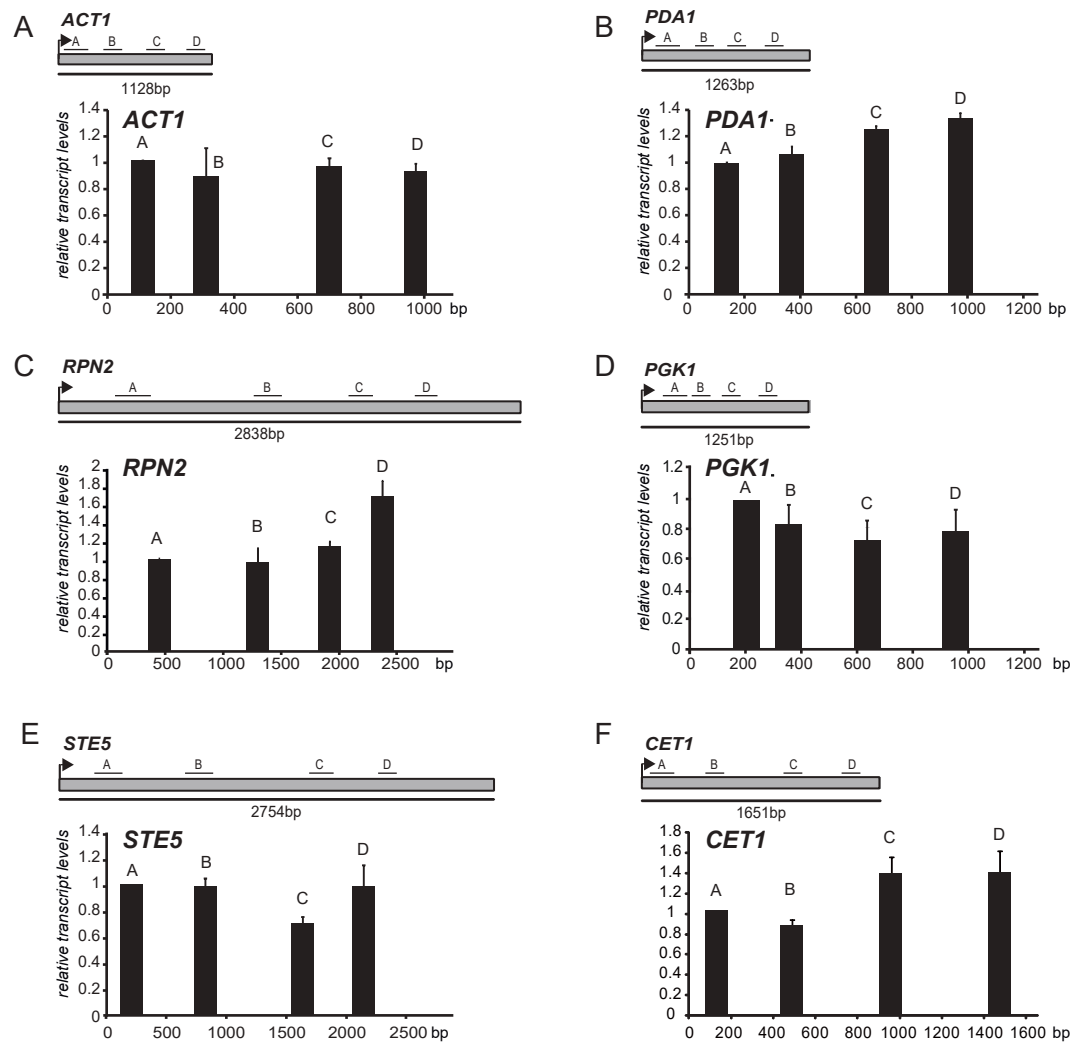

RAP containing genes without reduced transcript levels

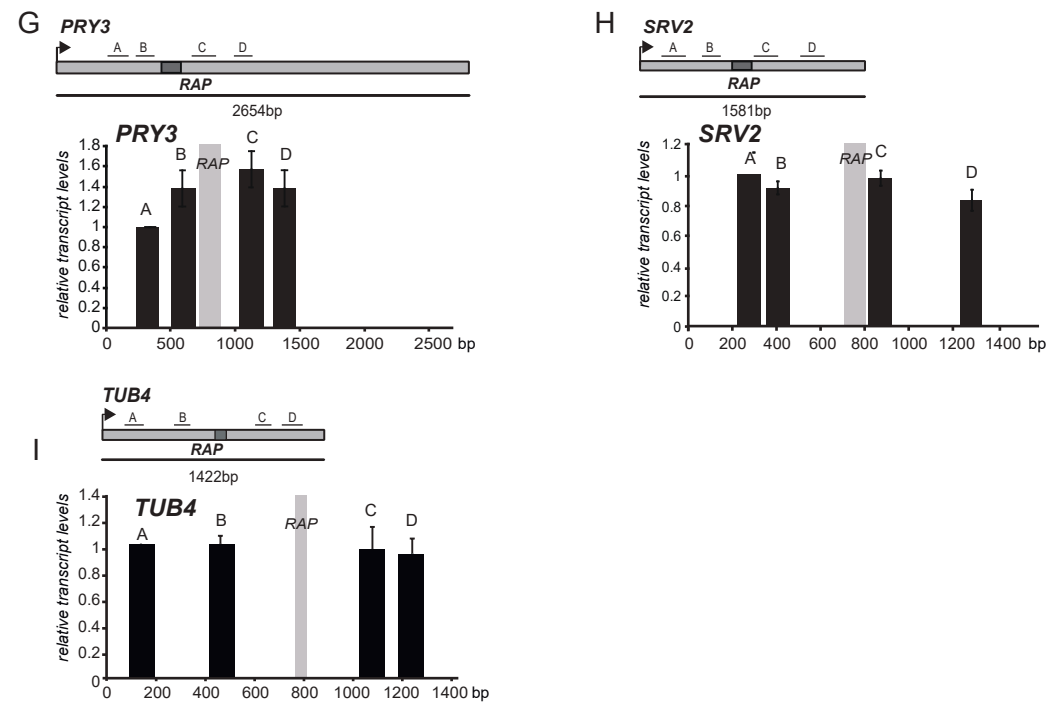

Supplement: S4 Fig — (A) to (F) Transcript levels of 6 selected target genes (ACT1, PDA1, RPN2, PGK1, STE5 and CET1) not containing RAPs. Reverse transcription was performed with random nonamers and expression levels were quantified by qRT-PCR using amplicons as indicated in the respective figures. Values were normalized to the first 5’ amplicon. (G) to (I) RAP-containing genes PRY3, SRV2 and TUB4 do not show any drop of transcript levels downstream of the RAPs. (PDF) [file pone.0194438.s004.pdf]

Supplementary Figure 5

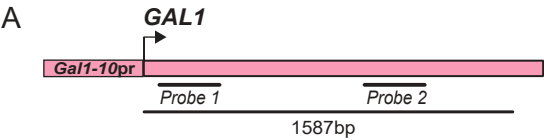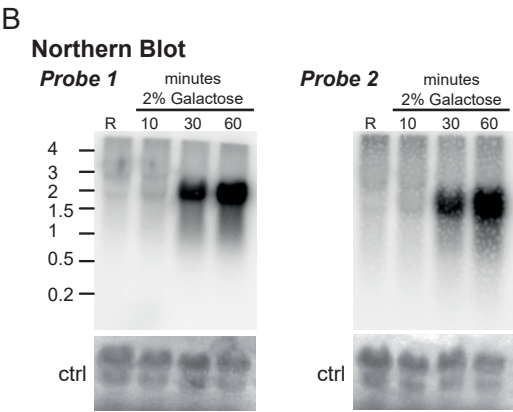

Supplement: S5 Fig — (A) Schematic of position of Northern Blot probes at the GAL1 locus under the control of its endogenous GAL1-10 promoter. (B) Northern Blot using the probes indicated in A). (PDF) [file pone.0194438.s005.pdf]

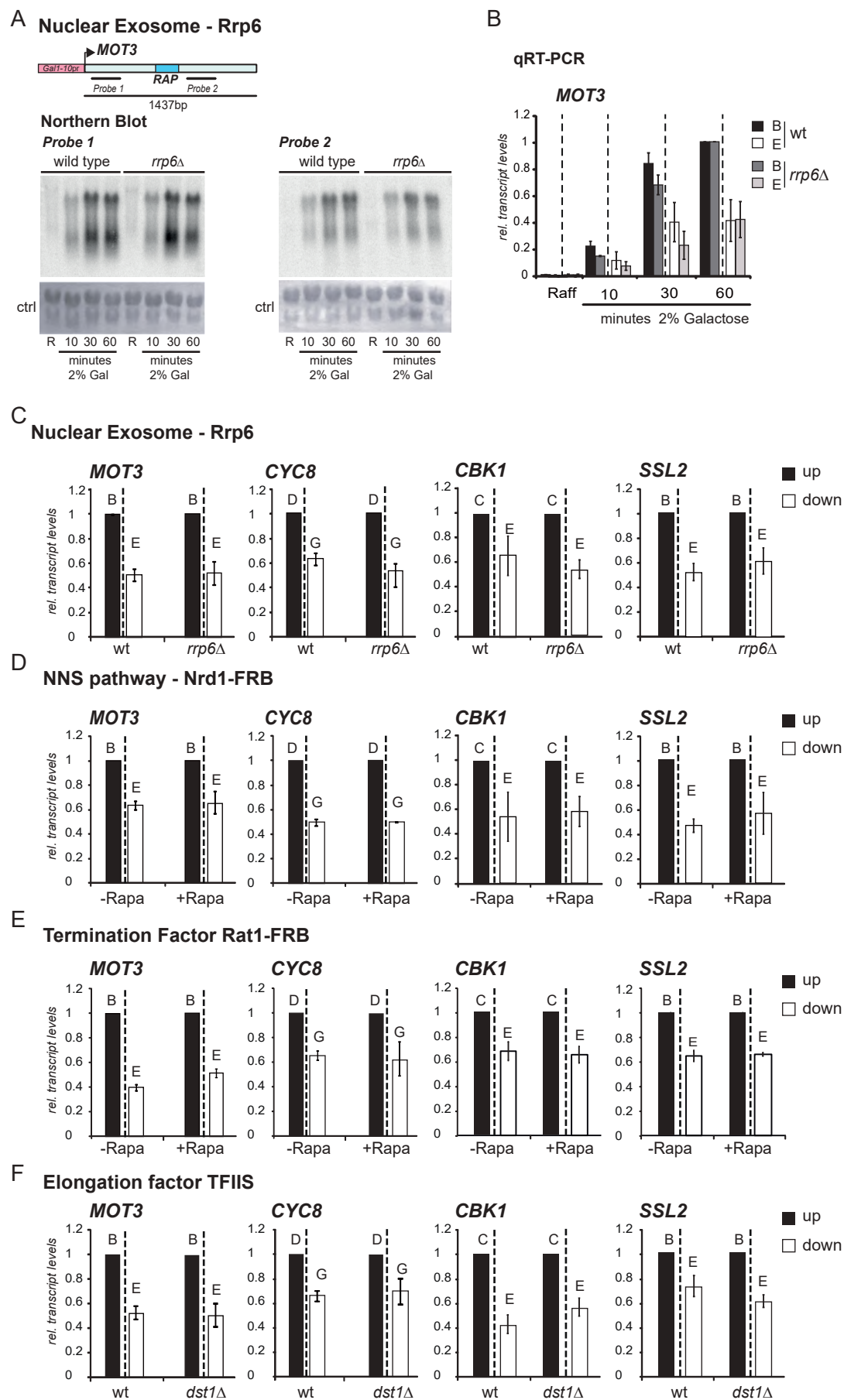

Supplement: S7 Fig — (A) Northern Blot as presented in Fig 3C but here also the nuclear exosome mutant rrp6Δ was analyzed. The short unstable transcripts detected with the upstream probe1 are similarly expressed in wild type and mutant strain. (B) qRT-PCR analyzing the same samples used for Northern Blotting show similar expression patterns in wild type and rrp6Δ mutant. (C) to F) qRT-PCRs of MOT3, CYC8, CBK1 and SSL2 wild type and mutant cells. Reverse transcription of DNA-free RNA was performed using random nonamers and quantification was done using primers upstream and downstream (B and E for MOT3, D and G for CYC8, C and E for CBK1, B and E for SSL2) of the RAP similar as in Fig 3) Nuclear exosome mutant lacking the intranuclear subunit Rrp6. (D) Mutant of the NNS-pathway component Nrd1. Nrd1-FRB cells were treated with 1μg rapamycin for 1 hour or left untreated. (E) Mutant of polyA-dependent termination pathway endonuclease Rat1. Rat1-FRB cells were treated with 1μg rapamycin for 1 hour or left untreated. (F) Mutant lacking the elongation factor Dst1, a component of TFIIS. (PDF) [file pone.0194438.s007.pdf]

Supplementary Figure 8

RNA Pol II Distribution at RAP-containing genes

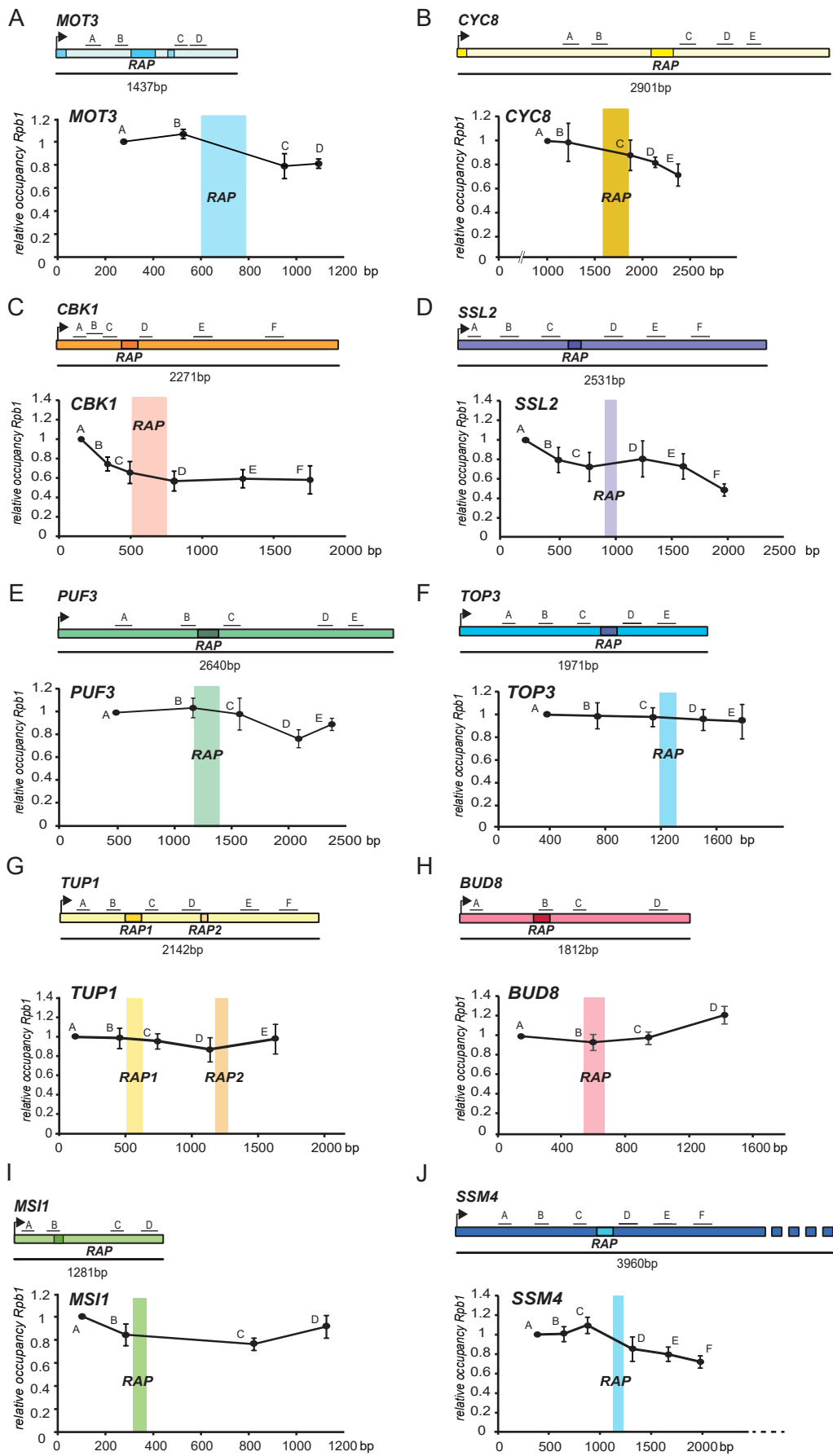

Supplement: S8 Fig — (A) to (J) Chromatin Immunoprecipitation of RNA Pol II (Rpb1) at RAP-containing target genes. Similar amplicons as for analysis of expression profiles were used (see Fig 2) for qRT-PCR. The majority of the analyzed loci show more RNA Pol II binding upstream of the RAP (except BUD8 and TUP1). The MOT3 and SSM4 loci seem to have a pause site immediately upstream of the RAP. (PDF) [file pone.0194438.s008.pdf]

Supplementary Figure 9

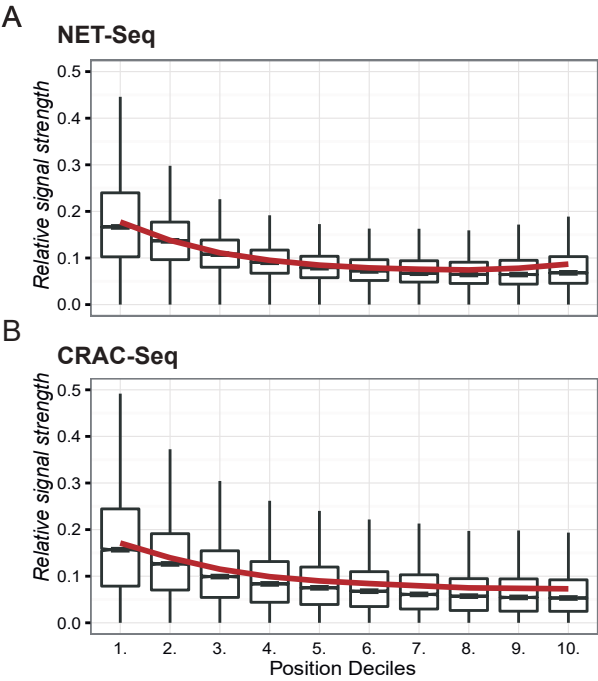

Supplement: S9 Fig — (A) Relative RNA Pol II occupancy across the gene body as seen in the CRAC-seq and NET-seq data (26,47). All the positions in all the annotated genes are bin into ten deciles, so that the first 10% of the gene bases are grouped in the 1st decile. The read signal in each decile is further normalized with the total read signal for each gene. Both data sets show that the average RNA Pol II occupancy is highest at the 5’ end of ORFs. (PDF) [file pone.0194438.s009.pdf]
