# Supplementary material for: Nascent RNA signaling to yeast RNA Pol II during transcription elongation
Source: PLoS One. 2018 Mar 23;13(3):e0194438. doi: 10.1371/journal.pone.0194438 (PMC5865726; doi:10.1371/journal.pone.0194438)
Supplement: S6 Fig — (A) to (C) 3’RACE results for MOT3, CBK1 and PUF3 under the control of the inducible GAL1-10 promoter. Positions of the RAPs and the used gene specific forward primers are indicated. PCR products resulting from amplification with the gene specific primer and the adapter specific primers are shown below the sequences. For MOT3 the transcripts lengths range from ~200-1100bp with a forward primer starting at 186bp, the majority having a size of 500bp. For PUF3 the lengths range from ~100-1000bp when forward primer 127fw is used and from 100–750 when primer 565fw is used. CBK1 shows a size distribution of 100-500bp with primer 81fw. The PCR products were cloned into the p-GEM vector (Promega) and 7–11 clones were sent for sequencing to confirm specificity. The frequency of the identified 3’ends of these clones are indicated. (PDF) [file pone.0194438.s006.pdf]

Supplementary Figure 6

A

MOT3 Chromosome XIII 409154..410626

ATGAATGCGGACCATCACCTGCAACAGCAGCAGCAACAGCGACAACAGCATCAACAACAACAGCATCAACAACAACAGCATCAGCATCAGCATCAACAGCAGCAGCACACGATATT
ACAAAATGTGTCGAACACTAACAATATCGGCAGCGATTCGCTGGCGTCACAGCCTTTCAACACGACTACT**GTTTCCTCTAACAAGGACGACG**TTATGGTGAACTCTGGGGCAAGAGA
ACTTCCAATGCCCTTACATCAGCAGCAGTATAT**ATAC**CCTTACTATCAGTATACAAG**TAAT**AACAGTAACAA**CAAT**AATGTGACGGCTGGTAACAATATGT**CTGC**GTCGCCGATTGTCC
ATAACAACAG**CAAC**AACAGCAACAACAGCAATATTTCTGCTTCTGATTACACTGTCGCAAACAACAGTACTAGCAA**TAAT**AACAATAA**TAAT**AATAATAACAACAATAATAACAATAA
TATTCACCCAAAC**CAGT**TTACTGCGGCCGCAAATATGAACTCAAATGCTGCAGCGGCTGCTTATTACTCCTTCCCCACTGCGAATATGCCAATACCGCAACAGGATCAACAATATATGT
TCAATCCTGCTTCATACATAAGCCATTACTATTCAGCAGTTAACAGCAATAACAATGGTAATAACGCCGCTAACAATGGCAGCAACAACTCTTCTCACTCAGCCCCAGCCCCGGCCCCC
GGTCCACCCCATCACCATCACCATCATAGTAATACACACAACAACCTCAACAATGGTGGTGCTGTAAATACAAACAACGCTCCTCAGCACCATCCAACGATAATAACGGATCAATTTCA
ATTCCAACTACAACAAAACCCTTCTCCAAATTTGAATCTCAATATTAACCCGGCACAACCTCTGCATCTACCTCCTGGTTGGAAAATAAACACTATGCCGCAACCACGTCCTACGACAGC
ACCTAACCATCCCCCTGCGCCGGTGCCTTCTTCGAACCCTGTGGCCTCGAACTTGGTTCCTGCCCCATCATCAGACCATAAATATATCCATCAATGCCAATTTTGTGAGAAGTCTTTCAA
AAGAAAATCATGGTTGAAAAGGCACCTATTGTCACACTCGCAACAAAGACATTTTCTATGCCCTTGGTGCTTAAGCAGGCAGAAGAGAAAAGATAATCTTTTACAGCATATGAAACTC
AAGCATACAAATTATTTATTAGACGAACTCAAGAAAAACAACATCATCTTTAACTACAACAATTCTTCCTCCTCTAATAATAACAACGACAATAATAATAATAATAACAGCAATAGCGC
TAGCGGCAGTGGCGGTGCCGGTGCCGCGGCGGCAGCAGCAACAGCTCCCGAAAATGAAGATGGAAACGGTTACGATACAAACATCAAGACTTTAATCAATGATGGTGTACTGAATA
AGGACGACGTTAAACGTGTTTTGAATAACCTTATTGTTAGTCACAACAAATAG

Primer MOT3 186fw: GTTTCCTCTAACAAGGACGACG

RAP 1018 | RAP 1028

RAP 1027 | RAP 1029

PCR product

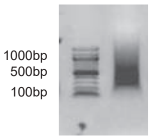

ATAC 1x
TAAT 1x
CAAT 1x
CTGC 1x
CAAC 1x
TAAT 4x
TAAT 1x
CAGT 1x
identified 3' ends

B

CBK1 Chromosome XIV 332597..334867

ATGTATAATAGCAGCACCAATCATCATGAGGGTGCACCTACTTCAGGACATGGCTATTATATGTCCCAACAACAAGACCAA**CAGCATCAACAACAACAACAATACGC**CAACGAAATG
AATCCGTATCAGCAAATTCCTAGACCGCCTGCTGCAGGATTTAGTAGCAACTACATGAAAGAGCAAGGCTCTCATCAATCGTTACAAGAGCATTT**ACAA**CGTGAGACAGGTAACCTT
GGCAGCGGTTTTACAGACGTTCCAGCCCTGAATTATCCAGCCACACCACCACCACATAA**TAAT**TACGCAG**CTTC**AAATCAGATGAT**CAAT**ACCCCACCGCCGTCGATGGGTGGTCTTT
ATAGACA**TAAT**AATAATTCCCAGAGCATGGTTCAGAACGGTAATGGGAGTGGGAATGCACAATTACCCCAATTA**TCAC**CGGGCCAATATTCTATAG**AGTC**AGAGTACAATCAAAATT
TAAATGGCAGTTCTTCTAGTAGTCCGTTCCACCAACCGCAAACGCTACGGAGCAATGGGTCATATTCAAGCGGGTTAAGGAGTGTGAAAAGCTTCCAGAGGTTACAACAAGAACAG
GAAAATGTCCAAGTTCAGCAACAACTTTCCCAAGCACAGCAACAAAATTCCCGGCAACAACAGCAACAACTTCAATACCAGCAGCAGCAACAACAACAACAACAACAGCAACATATG
CAAATACAACAACAACAACAGCAGCAACAACAGCAGCAGCAATCACAATCTCCCGTTCAGAGCGGCTTTAATAATGGTACCATATCAAACTATATGTACTTTGAAAGACGACCAGATT
TGTTGACTAAAGGAACGCAAGATAAAGCAGCAGCTGTAAAGTTGAAGATTGAGAACTTTTACCAATCCTCTGTTAAATACGCCATTGAAAGAAATGAAAGAAGAGTGGAGTTAGAA
ACAGAACTAACTTCTCATAATTGGTCAGAAGAAAGGAAATCTAGGCAATTGAGTTCTTTGGGTAAAAAAGAGTCACAATTTTTAAGGCTGCGTAGAACGAGATTATCGTTAGAAGAT
TTCCACACTGTGAAAGTCATTGGAAAAGGGGCATTTGGTGAAGTCAGACTGGTTCAGAAGAAAGACACCGGAAAAATATATGCAATGAAAACTTTATTAAAATCCGAAATGTACAAA
AAGGACCAATTAGCACACGTCAAGGCTGAAAGGGATGTTCTGGCTGGAAGTGATTCTCCATGGGTGGTTTCGCTATATTACTCATTCCAAGATGCTCAATACTTATATTTAATCATGG
AATTTTTGCCCGGTGGTGATTTGATGACCATGCTAATCAGGTGGCAACTATTTACAGAGGATGTTACTAGATTTTACATGGCTGAGTGTATTTTGGCCATCGAAACCATTCATAAATTA
GGATTCATTCACAGAGATATTAAACCAGATAATATTTTGATCGATATTAGAGGTCATATAAAATTATCTGATTTCGGGTTGTCTACGGGGTTCCATAAAACTCACGACTCAAACTACTA
TAAAAAGCTTTTGCAACAAGATGAGGCAACTAATGGTATTTCCAAGCCAGGTACTTACAATGCAAATACAACCGATACTGCAAATAAAAGGCAAACAATGGTTGTGGATTCTATTAGT
CTAACAATGTCAAACAGGCAACAAATTCAGACATGGAGAAAATCACGTCGTTTAATGGCATATTCTACCGTAGGTACACCAGATTATATTGCTCCTGAGATATTTTTATACCAAGGTTA
TGGGCAAGAGTGTGATTGGTGGTCACTTGGAGCAATTATGTACGAGTGTCTGATTGGTTGGCCTCCATTCTGTTCCGAAACTCCACAGGAAACGTACAGAAAAATCATGAATTTTGA
GCAAACTCTACAATTTCCTGATGATATTCATATCTCTTATGAAGCCGAAGATCTAATCCGTCGACTATTAACTCATGCAGACCAAAGACTTGGTAGACATGGTGGTGCAGATGAAATA
AAAAGTCATCCATTTTTTCGCGGTGTTGATTGGAATACAATCAGACAAGTAGAAGCACCGTATATACCAAAGTTGAGCTCCATCACCGATACTAGGTTCTTCCCAACCGATGAGTTGG
AGAATGTTCCAGATTCCCCAGCTATGGCACAAGCTGCCAAACAAAGGGAACAGATGACAAAGCAAGGTGGAAGTGCGCCAGTCAAGGAAGATTTACCTTTTATTGGCTACACTTACT
CCAGATTTGACTATTTGACAAGAAAAAATGCGTTGTAG

PCR product

Primer CBK1 81fw: CAGCATCAACAACAACAACAATACGC

RAP 1105

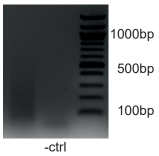

ACAA 1x
TAAT 1x
CTTC 3x
CAAT 1x
TAAT 2x
TCAC 1x
AGTC 1x
identified 3' ends

C

PUF3 Chromosome XII 122075..124714

ATGGAAATGAACATGGATATGGATATGGATATGGAACTCGCATCCATAGTTTCTTCTTTAAGCGCCCTGTCCCATAGTAACAACAACGGCGGTCAGGCCGCCGCGGCCGGCATTGTG
AACGGCGGTG**CTGCTGGATCACAGCAAATTG**GCGGCTTCAGAAGGTCGAGTTTCACCACGGCAAATGAAGTAGATTCAGAGATCTTGCTGCTTCACGGTTCCAGTGAATCTTCACCG
ATCTTCAAGAAAACCGCCCTATCAGTTGGTACTGCTCCGCCATTCTCCAC**GAAC**TCGAA**AAAG**TTTTTTGGTAACGGCGGCAATTATTACCAGTACAGATCAACCGACACTGCCTCCCT
GTCATCCGCATCGTACAATA**ACTA**TCATACTCATCATACTGCGGCTAATCTCGGGAAAAACAACAAGGTCAACCATCT**CTGG**GCCAGTATTCAGCTTCCATTGCTGGTCCCGTTTACTA
CAATGGCAATGACAACA**ACAA**CTCTGGTGGGGAAGGGTTTTTTGAGAAGTTCGGAAAATCTTTGATTGACGGTACAAGGGAACTGGAATCTCAAGATAGACC**CGACGCTGTCAATA**
CTCAGTCCCAATTCATATCAAAAAGCGTCTCTAACGCCAGCTTAGACACACAGAATACTTTCGAGCAAAATGTCGAGTCTGACAAAAA**CTTC**AAC**AAAT**TGAATAGGAACACTACTAA
CAGTG**GTTC**GCTTTACCACTCCAGTTCAAATTCAGGCTCATCTGCGTCTTTAGAGTCCGAAAATGCTCATTACCCCAAGAGAAATATCTGGAACGTAGCCAACACACCA**GTAT**TTAGAC
CTA**GTAA**CAATCCAGCCGCTGTTGGGGCCACAAATGTCGCGCTTCCTAACCA**ACAA**GATGGGCCTGCAAATAACAATTTCCCCCCCTACATGAACGGCTTCCCACCCAATCAGTTTCA
TCAGGGCCCTCATTACCAAAACTTCCCCAACTACTTGATCGGATCTCCTTCAAATTTCATCTCCCAGATGATTTCAGTACAGATTCCAGCAAATGAAGACACCGAAGACTCAAATGGTA
AGAAAAAGAAGAAGGCAAACAGACCTTCTTCTGTTTCTTCACCTTCGTCCCCTCCAAACAATTCTCCGTTCCCGTTTGCCTACCCCAACCCAATGATGTTTATGCCACCTCCACCACTCTC
TGCTCCCCAGCAACAACAGCAACAGCAACAACAACAACAACAAGAAGACCAACAACAGCAACAGCAACAGGAGAATCCTTATATTTACTACCCTACTCCAAATCCTATACCTGTCAAA
ATGCCCAAGGACGAAAAAACCTTCAAGAAAAGAAATAACAAGAATCATCCTGCAAATAACTCCAACAACGCCAACAAACAGGCAAATCCTTATCTAGAAAATTCTATACCCACAAAG
AATACCTCTAAAAAGAATGCTTCTTCCAAATCTAATGAGTCAACGGCCAACAACCATAAATCTCACTCTCATTCTCATCCTCATTCTCAAAGTCTACAGCAACAGCAGCAAACGTATCAT
CGATCGCCGTTGTTAGAGCAACTAAGAAACAGTAGTTCTGACAAAAATTCAAACTCGAACATGTCACTAAAAGACATCTTTGGCCATTCCTTAGAGTTTTGCAAAGATCAACATGGTT
CACGGTTCATTCAGCGCGAGTTGGCCACCTCACCAGCCTCAGAAAAGGAGGTCATATTCAATGAAATTCGCGATGACGCCATTGAGCTTTCGAACGATGTATTTGGTAATTACGTCAT
TCAGAAGTTTTTTGAATTTGGTAGCAAAATTCAAAAAAATACTTTGGTTGATCAATTTAAAGGTAACATGAAACAGTTATCTCTACAAATGTACGCATGTCGTGTAATTCAAAAGGCGT
TAGAATATATTGATTCCAATCAAAGAATTGAGTTAGTCCTGGAATTATCTGACTCCGTTTTGCAAATGATCAAAGATCAGAACGGTAATCACGTAATTCAAAAGGCCATCGAAACAAT
TCCTATCGAAAAATTACCTTTTATCTTAAGTTCTTTAACTGGCCACATTTACCATCTATCTACCCACTCCTACGGCTGTAGGGTCATTCAAAGATTGTTAGAATTTGGTTCGAGCGAAGA
TCAGGAAAGCATTTTAAACGAATTGAAAGATTTCATACCATATCTAATTCAAGACCAATATGGTAACTATGTCATTCAATATGTTTTGCAACAAGATCAGTTCACCAATAAGGAAATGG
TCGATATCAAACAGGAAATCATAGAAACTGTGGCCAATAATGTTGTCGAATACTCTAAACATAAATTTGCCTCCAACGTGGTCGAAAAATCTATTCTTTATGGCTCTAAAAATCAGAAG
GATTTAATAATTTCTAAGATTTTACCAAGAGACAAAAATCATGCTTTGAATCTGGAAGACGATTCTCCAATGATTTTAATGATCAAGGATCAATTTGCCAACTATGTGATTCAAAAATT
AGTTAATGTCTCTGAGGGTGAGGGTAAGAAATTGATCGTCATTGCCATTAGAGCATATTTGGATAAACTAAACAAATCTAACTCCCTAGGAAATAGACATTTAGCCAGTGTTGAGAA
ACTTGCAGCATTGGTTGAAAATGCGGAGGTGTGA

PCR products

Primer PUF3_127fw: CTGCTGGATCACAGCAAATTG

Primer PUF3_565fw: CGACGCTGTCAATACTCAGTC

RAP 809

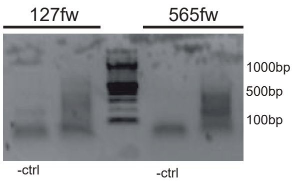

GAAC 1x
AAAG 3x
ACTA 1x
CTGG 1x
ACAA 1x
identified 3' ends 127fw

CTTC 2x
AAAT 2x
GTTC 1x
GTAT 1x
GTAA 2x
ACAA 1x
identified 3' ends 565fw
