# Supplementary material for: Nascent RNA signaling to yeast RNA Pol II during transcription elongation
Source: PLoS One. 2018 Mar 23;13(3):e0194438. doi: 10.1371/journal.pone.0194438 (PMC5865726; doi:10.1371/journal.pone.0194438)
Supplement: S2 Table — (DOCX) [file pone.0194438.s011.docx]

**Table S2 RAP containing target genes**

| ***Gene*** | ***Function*** | ***RAP*** | ***Sequence*** | ***Length*** | ***Read Count*** | ***Effect*** |
| --- | --- | --- | --- | --- | --- | --- |
| YMR070W | Transcriptional repressor, activator; role in cellular adjustment to osmotic stress, including transcriptional activation and modulation of mating efficiency; involved in repression of subset of hypoxic genes by Rox1p, repression of several DAN/TIR genes during aerobic growth, ergosterol biosynthetic genes in response to hyperosmotic stress; contributes to recruitment of Tup1p-Cyc8p general repressor to promoters; relocalizes to cytosol under hypoxia; can form [MOT3+] prion | *MOT3*  *RAP1018* | ACCUGCAACAGCAGCAGCAACAGCGACAACAGCAUCAACAACAACAGCAUCAACAACAACAGCAUCAGCAUCAGCAUCAAC | 81bp | 109 | 5‘ end |
|  |  | *MOT3*  *RAP1027* | AUACAUAAGCCAUUACUAUUCAGCAGUUAACAGCAAUAACAAUGGUAAUAACGCCGCUAACAAUGGCAGCAACAACUCUUCUCACUCAGCCCCAGCCCCGGCCCCC | 106bp | 53338 | 30-40% |
|  |  | *MOT3*  *RAP1028* | ACCCCAUCACCAUCACCAUCAUAGUAAUACACACAACAACCUCAACAAUGGUGGUGCUGUAAAUACAAACAACGCUCCUCAGCAC | 85bp | 199 | 30-40% |
|  |  | *MOT3*  *RAP1029* | CCUCUGCAUCUACCUCCUGGUUGGAAAAUAAACACUAUGCCGCAACCACGUCCUACGACAGCACCUAACCA | 71bp | 81 | no |
| YBR112C | General transcriptional co-repressor; acts together with Tup1p; also acts as part of a transcriptional co-activator complex that recruits the SWI/SNF and SAGA complexes to promoters; can form the prion [OCT+] | *CYC8*  *RAP133* | GGCGGUGAACAAACAAUAAUGGAACAACCCGCUCAACAGCAACAACAACAGCAACAACAACAGCAGCAACAGCAACAGCAGGCAGCAGUUCCUCAGCAGCCACUCGACCCAUUAACACAAUCAACUGCGGAAACUUGGCUCUCCAUUGCUUCUUUGGCAGAAACCCUUGGU | 163bp | 179 | ~40% |
|  |  | *CYC8*  *RAP134* | CGCAAGCACAGGCACAAGCACAAGCACAAGCACAUGCACAAGCGCAAGCACAAGCACAAGCACAGGCACAAGCACAAGCACAGGCGCAGGCACAACAACAACAACAACAACAGCAACAACAACAACAACAACAACAACAACAACAACAACAACAACAACAACAACAACAACAACAGCAGCAGCAAUUACAGCCCCUACCAAGACAACAGCUGCAGCAAAAGGGA | 106bp | 94 | 5’ end |
| YNL161W | Serine/threonine protein kinase of the the RAM signaling network; Ndr/LATS family member; binds regulatory subunit Mob2p; involved in regulation of cellular morphogenesis, polarized growth, and septum destruction; phosphorylation by Cbk1p regulates localization and activity of Ace2p transcription factor and Ssd1p translational repressor; Cbk1p activity is regulated by both phosphorylation and specific localization; relocalizes to cytoplasm upon DNA replication stress | *CBK1*  *RAP1105* | GAGUGUGAAAAGCUUCCAGAGGUUACAACAAGAACAGGAAAAUGUCCAAGUUCAGCAACAACUUUCCCAAGCACAGCAACAAAAUUCCCGGCAACAACAGCAACAACUUCAAUACCAGCAGCAGCAACAACAACAACAACAACAGCAACAUAUGCAAAUACAACAACAACAACAGCAGCAACAACAGCAGCAGCAAUCACAAUCUC | 206bp | 77 | 50-60% |
| YIL143C | Component of RNA polymerase transcription factor TFIIH holoenzyme; acts as a dsDNA-dependent translocase in the context of TFIIH, allowing unwinding of promoter DNA; Ssl2p alone has DNA-dependent ATPase/helicase activity; interacts functionally with TFIIB and has roles in transcription start site selection and in gene looping to juxtapose initiation and termination regions; involved in DNA repair; relocalizes to the cytosol in response to hypoxia; homolog of human ERCC3 | *SSL2*  *RAP319* | AGAAGAAGAUGAUGAUAUUGAUGCCGUUCACUCCUUUGAAAUUGCCAAUGAGUCUGUUGAAGUCGUAAAGAAA | 73bp | 2656 | ~40% |
| YLL013C | Protein of the mitochondrial outer surface; links the Arp2/3 complex with the mitochore during anterograde mitochondrial movement; also binds to and promotes degradation of mRNAs for select nuclear-encoded mitochondrial proteins | *PUF3*  *RAP809* | ACCCCAACCCAAUGAUGUUUAUGCCACCUCCACCACUCUCUGCUCCCCAGCAACAACAGCAACAGCAACAACAACAACAACAAGAAGACCAACAACAGCAACAGCAACAGGAGAAUCCUUAUAUUUACUACCCUACUCCAAAUCCUAUACCUGUCAAAAUGCCCAAGGACGAAAAAACCUUCAAGAAAAGAAAUAACAAGAAUCAUCCUGCAAAUAACUCCAACAACGCCAACAAACAGGCAAAUCCUUAU | 251bp | 741 | ~70% |
| YLR234W | DNA Topoisomerase III; conserved protein that functions in a complex with Sgs1p and Rmi1p to relax single-stranded negatively-supercoiled DNA preferentially; DNA catenation/decatenation activity is stimulated by RPA and Sgs1p-Top3p-Rmi1p; involved in telomere stability and regulation of mitotic recombination | *TOP3*  *RAP929* | UCCAACCCGAAAACACAAGUAACAAUAACAAGUUCAAGUUUCCACGAAGC | 50bp | 920 | 20-30% |
|  |  | *TOP3*  *RAP*930 | AUGACGACAAAGCGCAUCCACCAAUCCACCCCAUCGUAAGUCUGGGGCCUGAAGCAAA | 58bp | 2350 | 20-30% |
| YBR195C | Subunit of chromatin assembly factor I (CAF-1); chromatin assembly by CAF-1 affects multiple processes including silencing at telomeres, mating type loci, and rDNA; maintenance of kinetochore structure; deactivation of DNA damage checkpoint after DNA repair; chromatin dynamics during transcription; and repression of divergent noncoding transcription; Msi1p localizes to nucleus and cytoplasm and independently regulates the RAS/cAMP pathway via sequestration of Npr1p kinase | *MSI1*  *RAP152* | CAAGGUUUCCCUCCAAACACUUAGUAAAUGACAUCAGUAUUUUC | 44bp | 145 | 30-60% |
| YLR353W | Protein involved in bud-site selection; diploid mutants display a unipolar budding pattern instead of the wild-type bipolar pattern, and bud at the proximal pole; BUD8 has a paralog, BUD9, that arose from the whole genome duplication | *BUD8*  *RAP941* | UAUACUCAUCUUUGGCGAAUCGCGGGAACGACGAGUCAAAGAAUGGAACACCUCCACGUCCUACCUCAAUUGAACCUAAUGAAACAACGGAACACUCAUUUUUCUCAU | 109bp | 49 | 20-30% |
| YIL030C | Membrane-embedded ubiquitin-protein ligase; ER and inner nuclear membrane localized RING-CH domain E3 ligase involved in ER-associated protein degradation (ERAD); targets misfolded cytosolic/nucleoplasmic domains of soluble and membrane embedded proteins (ERAD-C) and a transmembrane domain containing substrate (ERAD-M), Sbh2p; C-terminal element (CTE), conserved in human ortholog MARCH10/TEB4, determines substrate selectivity | *SSM4 RAP354* | ACAGCAGCAACCAGAAGAAGAGGCAGAUUACCGUGAUCAUAUAGAGCCCAAUCCGAUUG | 59bp | 161 | ~50% |
| YCR084C | General repressor of transcription; forms complex with Cyc8p, involved in the establishment of repressive chromatin structure through interactions with histones H3 and H4, appears to enhance expression of some genes | *TUP1*  *RAP181* | AACGCCCAACAACAACUACCACAACAGCAACUGCAACAGCAGCAACUUCAACAACAGCAACCACCUCCCCAGGUUUCCGU | 80bp | 107 | No effect |
|  |  | *TUP1*  *RAP180* | ACCACCACGUCCACGGAUAACAAUACAAUGACAACCACUACUACCACCACAAU | 53bp | 49 | ~50% |
|  |  | *TUP1 RAP194** | GGUUUUCCACAUCUUUUGCCAAUUCUGCUGCCGAAGUCAUCGCUGUGGUAGUAAUUGUGGUGGUAGUAGUGGUUGUCAUUGUAUUGUUAUCCGUGGACGUGGUG | 104bp | 64 | antisense |
| YJL078C | Cell wall-associated protein involved in export of acetylated sterols; member of the CAP protein superfamily (cysteine-rich secretory proteins (CRISP), antigen 5, and pathogenesis related 1 proteins); role in mating efficiency; expression of full-length transcript is daughter cell-specific; in response to alpha factor, a short transcript starting at +452 is expressed and the long form is repressed by Ste12p | *PRY3*  *RAP694* | AGCCUCAAGCUCUUCUGUCACUACUUCCUAUGCUACCUCCUCGAGUACCGUCGUCUCUAGUGAUGCUACUUCAUCCACUACCACCACCUCAUCGGUUGCUACAUCGUCCAGUACCACUUCUUCCGACC | 128bp | 2011 | No effect |
| YNL138W | CAP (cyclase-associated protein); N-terminus binds adenylate cyclase and facilitates activation by RAS; N-terminus forms novel hexameric star-shaped shuriken structures that directly catalyze cofilin-mediated severing of actin filaments; C-terminus, in physically and genetically separate activity, binds and recycles cofilin bound, ADP-actin monomers, facilitating regulation of actin dynamics and cell morphogenesis; N- and C-termini can function as physically separate proteins | *SRV2 RAP1110* | CAAUCAACGAAGAAUACAGGUGCUACUUCAUCUCCUUCGCCAGCAAGUGCUACAGCGGCUCCAGCACCACCACCUCCUCCACCAGCCCCACCAGCUUCCGUCUUUGAAAUCUCUAAUGA | 119bp | 380 | No effect |
| YLR212C | Gamma-tubulin; involved in nucleating microtubules from both the cytoplasmic and nuclear faces of the spindle pole body; protein abundance increases in response to DNA replication stress | *TUB4*  *RAP865* | UUUACUCUACCUUGAUUCCUUCUCCUGAACUU | 32bp | 37 | No effect |
