# Supplementary material for: Nascent RNA signaling to yeast RNA Pol II during transcription elongation
Source: PLoS One. 2018 Mar 23;13(3):e0194438. doi: 10.1371/journal.pone.0194438 (PMC5865726; doi:10.1371/journal.pone.0194438)
Supplement: S3 Table — (DOCX) [file pone.0194438.s012.docx]

**Table S3 Strains used in this study**

| **Strains** | **Genotype** | **Reference or Source** |
| --- | --- | --- |
| BY wt *α* | *Mat α* *his3*Δ1 *leu2*Δ0 *met15*Δ0 *ura3*Δ0 | Euroscarf |
| BY *rrp6*Δ *α* | *Mat α* *his3*Δ1 *leu2*Δ0 *met15*Δ0 *ura3*Δ0 *rrp6*Δ::kanMX | Euroscarf |
| BY *dst1*Δ *α* | *Mat α* *his3*Δ1 *leu2*Δ0 *met15*Δ0 *ura3*Δ0 *dst1*Δ::kanMX | Euroscarf |
| Nrd1-FRB | *Mat α tor1-1 fpr1::natMX Rpl13A-2×Fkbp12::TRP1Nrd1-Frb::kanMX6* | P.Cramer |
| Rat1-FRB | *Mat α tor1-1 fpr1::natMX Rpl13A-2×Fkbp12::TRP1Rat1-Frb::kanMX6* | This study |
| **Reporter Strains** | | |
| EKYGH | *Mat α* *his3*Δ1 *leu2*Δ0 *met15*Δ0 *ura3*Δ0 natMX::*GAL1-10prGFPHIS3* | This study |
| EKYGUH | *Mat α* *his3*Δ1 *leu2*Δ0 *met15*Δ0 *ura3*Δ0 natMX::*GAL1-10prGFPURA3STOPHIS3* | This study |
| EKYGCH | *Mat α* *his3*Δ1 *leu2*Δ0 *met15*Δ0 *ura3*Δ0 natMX::*GAL1-10prGFPRAPCYC8HIS3* | This study |
| EKYGCAAH | *Mat α* *his3*Δ1 *leu2*Δ0 *met15*Δ0 *ura3*Δ0 natMX::*GAL1-10prGFPRAPCAAHIS3* | This study |
| EKYGAACH | *Mat α* *his3*Δ1 *leu2*Δ0 *met15*Δ0 *ura3*Δ0 natMX::*GAL1-10prGFPRAPAACHIS3* | This study |
| EKYGACAH | *Mat α* *his3*Δ1 *leu2*Δ0 *met15*Δ0 *ura3*Δ0 natMX::*GAL1-10prGFPRAPACAHIS3* | This study |
| EKYGCCAH | *Mat α* *his3*Δ1 *leu2*Δ0 *met15*Δ0 *ura3*Δ0 natMX::*GAL1-10prGFPRAPCCAHIS3* | This study |
| EKYGTTGH | *Mat α* *his3*Δ1 *leu2*Δ0 *met15*Δ0 *ura3*Δ0 natMX::*GAL1-10prGFPRAPTTGHIS3* | This study |
| EKYGGGTH | *Mat α* *his3*Δ1 *leu2*Δ0 *met15*Δ0 *ura3*Δ0 natMX::*GAL1-10prGFPRAPGGTHIS3* | This study |
| EKYGCAGH | *Mat α* *his3*Δ1 *leu2*Δ0 *met15*Δ0 *ura3*Δ0 natMX::*GAL1-10prGFPRAPCAGHIS3* | This study |
| EKYGGAAH | *Mat α* *his3*Δ1 *leu2*Δ0 *met15*Δ0 *ura3*Δ0 natMX::*GAL1-10prGFPRAPGAAHIS3* | This study |
| EKYGTELCAH | *Mat α* *his3*Δ1 *leu2*Δ0 *met15*Δ0 *ura3*Δ0 natMX::*GAL1-10prGFPRAPTELCAHIS3* | This study |
| EKYGTELGUH | *Mat α* *his3*Δ1 *leu2*Δ0 *met15*Δ0 *ura3*Δ0 natMX::*GAL1-10prGFPRAPTELGUHIS3* | This study |
| EKYG201H | *Mat α* *his3*Δ1 *leu2*Δ0 *met15*Δ0 *ura3*Δ0 natMX::*GAL1-10prGFPRAP201HIS3* | This study |
| EKYG201rcH | *Mat α* *his3*Δ1 *leu2*Δ0 *met15*Δ0 *ura3*Δ0 natMX::*GAL1-10prGFPRAP201rcHIS3* | This study |
| **Overexpressed RAP-containing targets** | | |
| Gal1prMOT3kan | *Mat α* *his3*Δ1 *leu2*Δ0 *met15*Δ0 *ura3*Δ0 kanMX::*GAL1-10prMOT3* | This study |
| Gal1prMOT3 rrp6 | *Mat α* *his3*Δ1 *leu2*Δ0 *met15*Δ0 *ura3*Δ0 natMX::*GAL1-10prMOT3 rrp6*Δ::kanMX | This study |
| Gal1prCBK1 | *Mat α* *his3*Δ1 *leu2*Δ0 *met15*Δ0 *ura3*Δ0 kanMX::*GAL1-10prCBK1* | This study |
| Gal1prPUF3 | *Mat α* *his3*Δ1 *leu2*Δ0 *met15*Δ0 *ura3*Δ0 kanMX::*GAL1-10prPUF3* | This study |
| Gal1prMOT3nat | *Mat α* *his3*Δ1 *leu2*Δ0 *met15*Δ0 *ura3*Δ0 natMX::*GAL1-10prMOT3* | This study |
| **Plasmids used in this study** | | |
| pFA6a-FRB-GFP-His3MX6 |  | Euroscarf |
| pFA6a-FRB-His3MX6 |  | Euroscarf |
| pFA6kanMXGal1pr |  | Euroscarf |
| pFA6a-FRB-KanMX6 |  | Euroscarf |
| pYM-N25 |  | Euroscarf |
| pRS313 |  | Euroscarf |
| pRS316 |  | Euroscarf |
