# Supplementary material for: Nascent RNA signaling to yeast RNA Pol II during transcription elongation
Source: PLoS One. 2018 Mar 23;13(3):e0194438. doi: 10.1371/journal.pone.0194438 (PMC5865726; doi:10.1371/journal.pone.0194438)
Supplement: S4 Table — (DOCX) [file pone.0194438.s013.docx]

**Table S4 Primers used in this study**

| **Primer Targets** | **Name** | **Sequence fw** | **Name** | **Sequence rev** |  |  |  |  |  |  |
| --- | --- | --- | --- | --- | --- | --- | --- | --- | --- | --- |
| **Endogenous Targets** |  |  |  |  |  |  |  |  |  |  |
| MOT3 pair A | MOT3_91fw | ATCAACAGCAGCAGCACACGAT | MOT3_208rev | CGTCGTCCTTGTTAGAGGAAAC |  |  |  |  |  |  |
| MOT3 pair B | MOT3_203fw | CGACGTTATGGTGAACTCTGGGG | MOT3_352rev | GGACAATCGGCGACGCAGACA |  |  |  |  |  |  |
| MOT3 pair C | MOT3_474fw | CACCCAAACCAGTTTACTGCGG | MOT3_579rev | TTGATCCTGTTGCGGTATTGGC |  |  |  |  |  |  |
| MOT3 pair D | MOT3_922fw | TGCCGCAACCACGTCCTACG | MOT3_1024rev | GGTCTGATGATGGGGCAGGAACC |  |  |  |  |  |  |
| MOT3 pair E | MOT3_1007fw | GTTCCTGCCCCATCATCAGACC | MOT3_1189rev | GCTTGAGTTTCATATGCTGTAA |  |  |  |  |  |  |
| CYC8 pair A | CYC8_126fw | CAATCAACTGCGGAAACTTGGC | CYC8_238rev | GCAGATGAGGGATTGAACTGTA |  |  |  |  |  |  |
| CYC8 pair B | CYC8_396fw | GCTTACAATGCCTATCAACAGG | CYC8_506rev | GCATAGTCGAGCGAACCATATCT |  |  |  |  |  |  |
| CYC8 pair C | CYC8_897fw | TACCATCTCGGTAGAGTGCATAT | CYC8_1067rev | GCTCTTGTGTACGCGTCTAAGG |  |  |  |  |  |  |
| CYC8 pair D | CYC8_1164fw | AAGCAAGCTGCAAGACTGGA | CYC8_1310fw | AAAATCACAGGAGGTGGGGC |  |  |  |  |  |  |
| CYC8 pair E | CYC8_1774fw | CAAGACAACAGCTGCAGCAAAA | CYC8_1893rev | TGTAGAAAATGGTTGCAGTTGG |  |  |  |  |  |  |
| CYC8 pair F | CYC8_2049fw | AGATACATGGAAGGTGCAATCC | CYC8_2209rev | GCTTCTTTACCTGTGGTTCAGCG |  |  |  |  |  |  |
| CYC8 pair G | CYC8_2295 | AACCAATCGCCAGCAGTAGT | CYC8_2399 | GGTTCCTGTGCGCCAATTAC |  |  |  |  |  |  |
| CYC8 pair H | CYC8_2478fw | CCAGAGTCATCTAGTGTCCAACC | CYC8_2614rev | CTTCGTCTTCTACAGGGCTTGC |  |  |  |  |  |  |
| CBK1 pair A | CBK1_81_fwd | CAGCATCAACAACAACAACAATACGC | CBK1 185_rev | CCTTGCTCTTTCATGTAGTTGC |  |  |  |  |  |  |
| CBK1 pair B | CBK1 270fw | CCAGCCACACCACCACCACATAA | CBK1 438rev | TTGGCCCGGTGATAATTGGGGTA |  |  |  |  |  |  |
| CBK1 pair C | CBK1415fw | TACCCCAATTATCACCGGGCCAA | CBK1 539rev | CTTGAATATGACCCATTGCTCCG |  |  |  |  |  |  |
| CBK1 pair D | CBK1755fw | CGTTCAGAGCGGCTTTAATAATGG | CBK1 853rev | CTGCTGCTTTATCTTGCGTTCCT |  |  |  |  |  |  |
| CBK1 pair E | CBK1 1220fw | TGATTCTCCATGGGTGGTTTCG | CBK1 1348rev | CCTCTGTAAATAGTTGCCACCTG |  |  |  |  |  |  |
| CBK1 pair F | CBK1 1701fw | GCATATTCTACCGTAGGTACACC | CBK1 1857rev | CGTTTCCTGTGGAGTTTCGGAAC |  |  |  |  |  |  |
| CBK1 pair G | CBK1 2054fw | ACCGTATATACCAAAGTTGAGC | CBK1 2183rev | CCTTGCTTTGTCATCTGTTCCCT |  |  |  |  |  |  |
| SSL2 pair A | SSL2_119_fwd | CTATGATGATAACAGGGAAACG | SSL2_272_rev | GCTTTATCCTTAGCGTCCATTTG |  |  |  |  |  |  |
| SSL2 pair B | SSL2_408_fwd | AGTGACGGTAGGATCATTCTGG | SSL2_570_rev | CGTCTCCAACCCTACAGAAACA |  |  |  |  |  |  |
| SSL2 pair C | SSL2_548fw | TGTTTCTGTAGGGTTGGAGACG | SSL2_720rev | ATCTGCTTGTGTAGTCTCCACG |  |  |  |  |  |  |
| SSL2 pair D | SSL2_1002fw | TATCCCGTGTTGGAAGAATACG | SSL2_1172rev | CCTGCGCCACATGGCAAAACAA |  |  |  |  |  |  |
| SSL2 pair E | SSL2_1233fw | ACTTCATCAGTCTCCGTCATGC | SSL2_1402rev | GAGACCTGTTTCTTGTGTTTGC |  |  |  |  |  |  |
| SSL2 pair F | SSL2_1899fw | CAAGAGCGTATGAACATTCTGC | SSL2_2039rev | CGAGACCCATAGTGCGAAGATA |  |  |  |  |  |  |
| PUF3 pair A | PUF3_127_fwd | CTGCTGGATCACAGCAAATTG | PUF3_325_rev | GGTAATAATTGCCGCCGTTACC |  |  |  |  |  |  |
| PUF3 pair B | PUF3_575_fwd | CGACGCTGTCAATACTCAGTC | PUF3_764_rev | GACTCTAAAGACGCAGATGAG |  |  |  |  |  |  |
| PUF3 pair C | PUF3_974_fwd | CTACTTGATCGGATCTCCTTC | PUF3_1135_rev | GGAACGGAGAATTGTTTGGAG |  |  |  |  |  |  |
| PUF3 pair D | PUF3_1455_fwd | GAGTCAACGGCCAACAACCA | PUF3_1661_rev | CGTGAACCATGTTGATCTTTGC |  |  |  |  |  |  |
| PUF3 pair E | PUF_1999_fwd | CCATCGAAACAATTCCTATCG | PUF_2142_rev | GCTTTCCTGATCTTCGCTCG |  |  |  |  |  |  |
| PUF3 pair F | PUF3_2278_fwd | CTGTGGCCAATAATGTTGTCG | PUF3_2438_rev | GGAGAATCGTCTTCCAGATTC |  |  |  |  |  |  |
| TOP3 pair A | TOP3_307_fwd | GCAACATCAAGCGAGAAGC | TOP3_480_rev | GTGTTGTCTTTCGAGATGC |  |  |  |  |  |  |
| TOP3 pair B | TOP3_612_fwd | CTGAGAAACCAAGCCACCAT | TOP3_818_rev | GTTGTTCCGCCGTTGTCTTTG |  |  |  |  |  |  |
| TOP3 pair C | TOP3_998_fwd | CCTGCGTCTGAACGCCAAAC | TOP3_1128_rev | CTTTTCGACCAAGGATTTTAGG |  |  |  |  |  |  |
| TOP3 pair D | TOP3_1317_fwd | CCAGTGGAAAGAAGAGTATACG | TOP3_1450_rev | CTACGAGACCTGAAGCTGAG |  |  |  |  |  |  |
| TOP3 pair E | TOP3_1527_fwd | ACTGAGAGTGAACTCATTC | TOP3_1730_rev | GGTTGTAAGTAGGTTTCCTTG |  |  |  |  |  |  |
| TUP1 pair A | TUP1 53fw | CATCAGACAGGAGTTTCTCCAAG | TUP1 201rev | CCTGTGAGTTAGTTCCAGTTCGT |  |  |  |  |  |  |
| TUP1 pair B | TUP1 388fw | AACAACCACCGGCTACTACTTCGG | TUP1 520rev | GCAAAGTGGTGGTAGGCAACTGTG |  |  |  |  |  |  |
| TUP1 pair C | TUP1 665fw | CACTACTTTACCCTCTGTCAAGG | TUP1 820rev | CGTCTTCCTCCTTAGGTTTGATTT |  |  |  |  |  |  |
| TUP1 pair D | TUP1 1062fw | GATGGTGAATACTTAGCCACAGG | TUP1 1205rev | GACGTGGTGGTGTTATTTTCAGT |  |  |  |  |  |  |
| TUP1 pair E | TUP1 1549fw | GCCAGTGTTCATTGACTTTATCC | TUP1 1701rev | CGAATCTAGTCTTTCCACCAAGA |  |  |  |  |  |  |
| TUP1 pair F | TUP1 1858fw | GCACTTGTGAAGTTACGTATATCG | TUP1 1992rev | CGGATTGCCGGATTTCTTATCCC |  |  |  |  |  |  |
| BUD8 pair A | BUD8_70fw | CCTCTGTATCATCCAGACTACA | BUD8_203rev | AATCCTTGACCTCTACTTGTGG |  |  |  |  |  |  |
| BUD8 pair B | BUD8_577fw | GGAACGACGAGTCAAAGAATGG | BUD8_684rev | TTCTAAGGTGTCATCGTAATGG |  |  |  |  |  |  |
| BUD8 pair C | BUD8_936fw | CCTCAGAGTCCATCGTTAATTG | BUD8_1100rev | GGTGGACCTTCTTCTTCTAGGG |  |  |  |  |  |  |
| BUD8 pair D | BUD8_1475fw | CGATACCGCTTCCATTCATTCG | BUD8_1617rev | CCTTCTTGAGCTGCAGCCAATA |  |  |  |  |  |  |
| MSI1 pair A | MSI1_27fw | TCATGAAGCCTCCAGTATACCC | MSI1_179rv | TCCGAAGTGGTATCTAAATCAGG |  |  |  |  |  |  |
| MSI1 pair B | MSI1_216fw | CCAAAAACCTGAAGATGAGACC | MSI1_359rv | ACTAAGTGTTTGGAGGGAAACC |  |  |  |  |  |  |
| MSI1 pair C | MSI1_756fw | AGCGGTGAATGATGTAACTTGG | MSI1_906rv | ACAGGAGTTTACTCCACCATCG |  |  |  |  |  |  |
| MSI1 pair D | MSI1_1053fw | TTTCGATACTGTATTGGCAACG | MSI1_1223rv | GAATTATCATTTGCCACACTGC |  |  |  |  |  |  |
| SSM4 pair A | SSM4_301fw | ACGATATATGCGGAAAACATGC | SSM4_451rv | TGTTCCATACCAAAGGAACACC |  |  |  |  |  |  |
| SSM4 pair B | SSM4_548fw | CTCCAGAATTAACCACGAGAGC | SSM4_717rv | TGGACCTATCTTGTGGAAAACC |  |  |  |  |  |  |
| SSM4 pair C | SSM4_791fw | ATTTAGCGAGGGAAATGAGAGC | SSM4_949rv | CAAAATTCCGATGGTCATTAGG |  |  |  |  |  |  |
| SSM4 pair D | SSM4_1224fw | TCAACAGAACGCAATAAACAGG | SSM4_1390rv | TTATCACTAGAGGGCCTTGTCC |  |  |  |  |  |  |
| SSM4 pair E | SSM4_1578fw | TATAGCTTACAATGGGCTGACG | SSM4_1741rv | ACGTCGTTAAAGCAGGTAAAGC |  |  |  |  |  |  |
| SSM4 pair F | SSM4_1912fw | CCCATTCTAGCTGGTGTAATGC | SSM4_2044rv | CAATCGTCCAGTATACGAAAAGG |  |  |  |  |  |  |
| SSM4 pair G | SSM4_2236fw | ATTGTCTTAGGTTTTGGCTTCC | SSM4_2399rv | CTAGACTCCAGGATCCTCTTCG |  |  |  |  |  |  |
| **Control Targets** |  |  |  |  |  |  |  |  |  |  |
| ACT1 pair A | ACT1_37_fwd | GTTCTGGTATGTGTAAAGCC | ACT1_192_rev | GATACCTCTCTTGGATTGAGC |  |  |  |  |  |  |
| ACT1 pair B | ACT1_390_fwd | GCCTTCTACGTTTCCATCCAAG | ACT1_561_rev | GTCAGTCAAATCTCTACCGGC |  |  |  |  |  |  |
| ACT1 pair C | ACT1_649_fwd | GTTACGTCGCCTTGGACTTCG | ACT1_796_rev | CAGAAGGATGGAACAAAGC |  |  |  |  |  |  |
| ACT1 pair D | ACT1_907_fwd | CCACCATGTTCCCAGGTATTG | ACT1_1068_rev | CCACATTTGTTGGAAGGTAGTC |  |  |  |  |  |  |
| PDA1 pair A | PDA1_70_fwd | CCACCAGGATAGGTCATGTTCG | PDA1_211_rev | CTGGAGGCTCTAGCATATACG |  |  |  |  |  |  |
| PDA1 pair B | PDA1_282_fwd | GAGATGGCTTGTGACGCCTTG | PDA1_432_rev | AGTGAAACCGTGACATCTGT |  |  |  |  |  |  |
| PDA1 pair C | PDA1_573_fwd | CCTTTAGGTGCAGGTTTAGCT | PDA1_742_rev | CGTACTTGTTGTTCTCACAGC |  |  |  |  |  |  |
| PDA1 pair D | PDA1_886_fwd | GCAAAGGTCCTCTCGTTCTA | PDA1_1083_rev | GGACTTGTCGTAAGCTTTGAC |  |  |  |  |  |  |
| RPN2 pair A | RPN2_340_fwd | CCAAGCAATACACTAAGGAC | RPN2_541_rev | CAGAAGTGGAATCCTGATCC |  |  |  |  |  |  |
| RPN2 pair B | RPN2_1174_fwd | GCAGTCGAGCTTCTTCTAGGT | RPN2_1353_rev | ACCGATACCCAGTGAAGCAC |  |  |  |  |  |  |
| RPN2 pair C | RPN2_1719_fwd | GTGGCAGTCTCGGATTCTAAC | RPN2_1883_rev | GCAATCCCAAGAGCAAATGC |  |  |  |  |  |  |
| RPN2 pair D | RPN2_2277_fwd | GGTATTCGTGGTAGCGATCAAG | RPN2_2439_rev | CCTTGCTTTTGCTCTTGCTG |  |  |  |  |  |  |
| PGK1 pair A | PGK1_126fw | GCTTTGCCAACCATCAAGTACG | PGK1_325rev | CCTTGACAGCGGCTTCAACTTC |  |  |  |  |  |  |
| PGK1 pair B | PGK1_256fw | CATTGTTGGGTAAGGATGTCACC | PGK1_404rev | CCATCGACCTTTCTGGAACCTT |  |  |  |  |  |  |
| PGK1 pair C | PGK1_576fw | AAGTACTTCGGTAAGGCTTTGGA | PGK1_761rev | GAGTCACCGATTTCAGTGTTTTC |  |  |  |  |  |  |
| PGK1 pair D | PGK1_838fw | TGCCAGTCGACTTCATCATTGC | PGK1_986rev | GCCTTTGCAACAGTAGCAGCAA |  |  |  |  |  |  |
| STE5 pair A | STE5_115fw | CCAGTACTCTATCCCCATTGTCA | STE5_300rev | CATTAGGTTACCGTCTTCATTGC |  |  |  |  |  |  |
| STE5 pair B | STE5_700fw | ACAAAGCCGTTCAATGCATTCC | STE5_927rev | TGCAGCCAATATGAGATTTGGG |  |  |  |  |  |  |
| STE5 pair C | STE5_1552fw | ATATCACTTCGACCCTGCCTATT | STE5_1751rev | CTCTGCCTAGAACATTCTCCTGA |  |  |  |  |  |  |
| STE5 pair D | STE5_2221fw | GCTATTTGAACGTTGACTACAG | STE5_2411rev | GCATCCGACAGTGATCCTGAAC |  |  |  |  |  |  |
| CET1 pair A | CET1 51fw | GATCTGGTGAATCACGATGAAA | CET1 198rev | CTTGTAATTGTCAATCGGAGCA |  |  |  |  |  |  |
| CET1 pair B | CET1 402fw | GCGAAGTTAGAAAAGCCTTCTG | CET1 550rev | TCTCAGTTTGCTTCTGCTTTTG |  |  |  |  |  |  |
| CET1 pair C | CET1 854fw | ACAGGACTGGGTTTATGCTACAA | CET1 1010rev | GCATCAAGCTCAGTGAAAACAC |  |  |  |  |  |  |
| CET1 pair D | CET1 1357fw | AAAGGACGAAAGACCGTGTTAG | CET1 1500rev | AGGCGTGTTGATTTCTAGTTCC |  |  |  |  |  |  |
| PRY3 pair A | PRY3_327fw | TCCACGGGTCACTTCACACAGGT | PRY3_451rev | GTAGTTTCCAGGAGGGTTGTAG |  |  |  |  |  |  |
| PRY3 pair B | PRY3_428fw | CTACAACCCTCCTGGAAACTACC | PRY3_546rev | GACTGTGTCTGATGTAGTTGAGG |  |  |  |  |  |  |
| PRY3 pair C | PRY3_1148fw | CGATATTCAGGGTAGCACTTCC | PRY3_1302rev | CGACACAGCTGAGGAAGAGACG |  |  |  |  |  |  |
| PRY3 pair D | PRY3_1280fw | CGTCTCTTCCTCAGCTGTGTCG | PRY3_1428rev | CGAAGTGATACTTTCTGAGGCG |  |  |  |  |  |  |
| SRV2 pair A | SRV2_162_fwd | GCCGATGCGAATACTACGAATG | SRV2_328_rev | GCAACTGCAAAGCATCTAAGACC |  |  |  |  |  |  |
| SRV2 pair B | SRV2_473_fwd | CCGTCAAAGCAAATACTTCGC | SRV2_585_rev | TGCGTCCTTGAAATCTGTGACC |  |  |  |  |  |  |
| SRV2 pair C | SRV2_892_fwd | CGAGTAGTGATGCTAACAAAGGC | SRV2_1041_rev | GGAAGAAACTGTAGAGGATTGACG |  |  |  |  |  |  |
| SRV2 pair D | SRV2_1151_fwd | CGAAAATGAAACTGAATCTCTGG | SRV2_1294_rev | GAACAACACTGCATGACTCAG |  |  |  |  |  |  |
| TUB4 pair A | TUB4_45fw | CCACGTTGGTAAGTTTCTCTGG | TUB4_217rv | GCTCGGAATCCATCATAATAGC |  |  |  |  |  |  |
| TUB4 pair B | TUB4_383fw | ATTCTACCGACAATTTCGAAGG | TUB4_524rv | GCAGGGAACACAGAATATGTCG |  |  |  |  |  |  |
| TUB4 pair C | TUB4_988fw | CAGATATCGCGTGCTATGACC | TUB4_1154rv | TTCACCACGGTAGACATATTGC |  |  |  |  |  |  |
| TUB4 pair D | TUB4_1149fw | GGTGAACGTCTTTGAGAATGC | TUB4_1318rv | CAGCTACATAATCCTCCATCAGG |  |  |  |  |  |  |
| **Reporter** |  |  |  |  |  |  |  |  |  |  |
| GFP A | GFP_345fw | GGTGATACCTTAGTTAATAGAATCG | GFP_541rev | GATGGTTCTGTTCAATTAGCTGACC |  |  |  |  |  |  |
| GFP B | GFP_565fw | GTGATGGTCCAGTCTTGTTACC | GFP_771rev | CGATGAATTCTCTGTCGGACCA |  |  |  |  |  |  |
| HIS3 C | HIS3_129fw | GCAGAACAGGCCACACAATCGC | HIS3_265rev | CGTCTATGTGTAAGTCACCAAT |  |  |  |  |  |  |
| HIS3 D | HIS3_389fw | GGTGGTAGATCTTTCGAACAGG | HIS3_593rev | GCAACCGCAAGAGCCTTGAACG |  |  |  |  |  |  |
| **Strain creation** |  |  |  |  |  |  |  |  |  |  |
| GalprMOT3 | F4_MOT3 fw | AAAGCCGACAGGGACAACGCGCGATAAAAAAAAAATTAAAAGTAAAACTAGAATTCGAGCTCGTTTAAAC | R2_MOT3 rev | TGATGCTGTTGTCGCTGTTGCTG  CTGCTGTTGCAGGTGATGGTCCGCATTCATCATTTTGAGATCCGGGTTTT |  |  |  |  |  |  |
| Gal1prPUF3 | F4_PUF3 fw | CATCTTCTTTCGTTCCTTCGCGTTTCTTTTCTCGTATATAATAAGCTTTTGCGAATTCGAGCTCGTTTAAAC | R2_PUF3 rev | GAAACTATGGATGCGAGTTCCATATCCATATCCATATCCATGTTCATTTCCATCATTTTGAGATCCGGGTTTT |  |  |  |  |  |  |
| Gal1prCBK1 | F4_CBK1 fw | CATATTATTACAATATAAAACTTTACGATAATTCCTGCAACTAATGATGCGAATTCGAGCTCGTTTAAAC | R2_CBK1 rev | TGTCCTGAAGTAGGTGCACCCTCATGATGATTGGTGCTGCTATTATACATCATTTTGAGATCCGGGTTTT |  |  |  |  |  |  |
| Rat1_Frb | Rat1-FRBfw | CAGCTACTCTCGGAATAACAAGCAAAGTCGGTATGACAATTCAAGAGCAAATAGGCGTCGGATCCCCGGGTTAATTAA | Rat1-FRBrev | GATTTTATAAATTTGCGAAAACCTAAATTTACCATAAAATAAAATGCGCACGAGTAGTTGAATTCGAGCTCGTTTAAAC |  |  |  |  |  |  |
| **Recombination Reporter** |  |  |  |  |  |  |  |  |  |  |
|  | natMXGal1prGFPfw | TGAAGGGCAGAGGAACAAGTGAAATACAACAGCTGAGTGCAGATAAGTGCCGCCAGATCTGTTTAGCTTGCC |  |  |  |  |  |  |  |  |
|  | GFPURA3fw | TCGATGATATCAGATCCACTAGTGGCCTATGCGGCCGCGGATCTGCCGGTCTCCCTAATGTCGAAAGCTACATATAAG |  |  |  |  |  |  |  |  |
|  | URAHIS3rev | AATCTTGGTTTCATTTGTAATACGCTTTACTAGGGCTTTCTGCTCTGTCATTTAGTTTTGCTGGCCGCATCTTC |  |  |  |  |  |  |  |  |
|  | GFPHIS3fw | TATCAGATCCACTAGTGGCCTATGCGGCCGCGGATCTGCCGGTCTCCCTA**atg**acagagcagaaagccctag |  |  |  |  |  |  |  |  |
|  | HIS3MAL13rev | CGCCCCTTTCTTATCAATGCTGAATACACCATTTCTAAGTAATATTATCACTACATAAGAACACCTTTGGTG |  |  |  |  |  |  |  |  |
