# Supplementary material for: Nascent RNA signaling to yeast RNA Pol II during transcription elongation
Source: PLoS One. 2018 Mar 23;13(3):e0194438. doi: 10.1371/journal.pone.0194438 (PMC5865726; doi:10.1371/journal.pone.0194438)
Supplement: S5 Table — (DOCX) [file pone.0194438.s014.docx]

| ***Name*** | ***Sequence*** | ***Length*** | ***Translated*** |
| --- | --- | --- | --- |
| ***CAA rich motif found in CYC8, CBK1 and PUF3*** | | | |
| *CYC8* full length | CAAGCACAAGCACAGGCGCAAGCACAGGCACAAGCACAAGCACAAGCACAUGCACAAGCGCAAGCACAAGCACAAGCACAGGCACAAGCACAAGCACAGGCGCAGGC**ACAACAACAACAACAACAACAGCAA**CAACAACAACAACAACAACAACAACAACAACAACAACAACAACAACAACAACAACAACAGCAGCAGCAAUUACAGCCCCUACCAAGACAACAGCUGCAGCAAAAGGGA | 240bp | QAQAQAQAQAQAQAQAHAQAQAQAQAQAQAQAQAQA**QQQQQQQQ**QQQQQQQQQQQQQQQQQQQQQQQLQPLPRQQLQQKG |
| CAA 15 | CAACAACAACAACAA |  |  |
| CAA hill | CAACAACAACAACAACAACAGCAA | 24bp | QQQQQQQQ |
| *UUG* | UUGCUGUUGUUGUUGUUGUUGUUG | 24bp | LLLLLLLL |
| *CAG* | **CAGCAGCAGCAGCAGCAGCAGCAG** | 24bp | QQQQQQQQ |
| *GAA* | **GAAGAAGAAGAAGAAGAAGAGGAA** | 24bp | EEEEEEEE |
| *AAC* | **AACAACAACAACAACAACAACAGCAA**A | 27bp | NNNNNNNSK |
| *ACA* | **ACAACAACAACAACAACAACAGCAA**AC | 27bp | UTTTTTTAN |
| ***Telomeric and Subtelomeric*** | | | |
| Telomere repeat 30bp C-rich | ACACACCACACCCACACCACACCCACACAC | 30bp | THHTHTTPTH |
| Telomere repeat 30bp G-rich | GGGUGUGGGUGUGUGGGUGUGGGUGUGGUG | 30bp | GCGCVGVGVV |
| Subtelomeric 201 | **CCACUCUAUACCACCACUACCACCACCGCCACUUGCCACACUCACCU**U | 48bp | PLYTTTTTTATCHTHL |
| Subtelomeric 201rc | AAGGUGAGUGUGGCAAGUGGCGGUGGUGGUAGUGGUGGUAUAGAGUGG | 48bp | KVSVASGGGGSGGIEW |

**Table S5 Sequences tested in the reporter system**
